# Use of Commercial Off-The-Shelf Devices for the Detection of Manual Gestures in Surgery: Systematic Literature Review

**DOI:** 10.2196/11925

**Published:** 2019-05-03

**Authors:** Fernando Alvarez-Lopez, Marcelo Fabián Maina, Francesc Saigí-Rubió

**Affiliations:** 1 Faculty of Health Sciences Universitat Oberta de Catalunya Barcelona Spain; 2 Faculty of Health Sciences Universidad de Manizales Caldas Colombia; 3 Faculty of Psychology and Education Sciences Universitat Oberta de Catalunya Barcelona Spain

**Keywords:** minimally invasive surgery, user-computer interface, operating room, education, medical, computer-assisted surgery

## Abstract

**Background:**

The increasingly pervasive presence of technology in the operating room raises the need to study the interaction between the surgeon and computer system. A new generation of tools known as commercial off-the-shelf (COTS) devices enabling touchless gesture–based human-computer interaction is currently being explored as a solution in surgical environments.

**Objective:**

The aim of this systematic literature review was to provide an account of the state of the art of COTS devices in the detection of manual gestures in surgery and to identify their use as a simulation tool for motor skills teaching in minimally invasive surgery (MIS).

**Methods:**

For this systematic literature review, a search was conducted in PubMed, Excerpta Medica dataBASE, ScienceDirect, Espacenet, OpenGrey, and the Institute of Electrical and Electronics Engineers databases. Articles published between January 2000 and December 2017 on the use of COTS devices for gesture detection in surgical environments and in simulation for surgical skills learning in MIS were evaluated and selected.

**Results:**

A total of 3180 studies were identified, 86 of which met the search selection criteria. Microsoft Kinect (Microsoft Corp) and the Leap Motion Controller (Leap Motion Inc) were the most widely used COTS devices. The most common intervention was image manipulation in surgical and interventional radiology environments, followed by interaction with virtual reality environments for educational or interventional purposes. The possibility of using this technology to develop portable low-cost simulators for skills learning in MIS was also examined. As most of the articles identified in this systematic review were proof-of-concept or prototype user testing and feasibility testing studies, we concluded that the field was still in the exploratory phase in areas requiring touchless manipulation within environments and settings that must adhere to asepsis and antisepsis protocols, such as angiography suites and operating rooms.

**Conclusions:**

COTS devices applied to hand and instrument gesture–based interfaces in the field of simulation for skills learning and training in MIS could open up a promising field to achieve ubiquitous training and presurgical warm up.

## Introduction

### Background

The increasingly pervasive presence of technology in the operating room raises the need to study the interaction between the surgeon and computer system. In sterile environments, using the hand to operate a mouse, keyboard, or touchscreen is unacceptable as it alters the normal pace of surgery and breaks asepsis and antisepsis protocols [1-6]. Using a physical barrier between the surgeon’s gloves and the interaction device [7], or the foot for manipulation, are not practical solutions either, as they do not allow fine interaction and carry risks of contamination [8]. Moreover, using a person to manipulate images in accordance with the surgeon’s verbal instructions has proven difficult and is prone to giving rise to misunderstandings when the visualization of specific areas of the image are requested [9,10].

Early solutions to circumvent any contact between the surgeon and computer were based on voice recognition Automated Endoscopic System for Optimal Positioning (AESOP) and HERMES (Stryker Europe) [11,12], but these systems were impractical as they were difficult to use when performing complex tasks [13]. Natural user interfaces were first developed in the 1990s to enable interaction with the computer through natural human movements to manipulate radiological images in sterile surgical environments [14]. Gesture-based interfaces were another variant [15]. These enabled touchless manipulations to be performed and held great promise as a viable solution in the operating room and autopsy suites [10,16-19]. However, they could not be employed in sterile environments as they required some contact when gloves or position sensors were used [20-24].

Early attempts to use touchless gestures in minimally invasive surgery (MIS) involved hand and facial gestures [9,25]. Gesture recognition systems with Web and video cameras were later described [26,27] using the time-of-flight principle [28] and achieving interaction with the OsiriX viewer [17,29]. However, these systems were very expensive and inaccurate and required calibration and a complex setup, making them impractical for use in the operating room [30].

A new generation of tools known as commercial off-the-shelf (COTS) devices enabling touchless gesture–based human-computer interaction is currently being explored as a solution in surgical environments. The term COTS refers to a device that can be taken from a shelf, that is, sold over the counter. In addition to being low-cost, wireless, and ergonomic, they facilitate real-time interactivity and allow the user to point to and manipulate objects with 6 degrees of freedom [31]. Hansen et al described the use of the Wii Remote (Nintendo) for the intraoperative modification of resection planes in liver surgery [32], whereas Gallo et al used it for pointing to and manipulating 3-dimensional (3D) medical data in a number of ways [31,33-36]. However, intraoperative manipulation of the device required it to be wrapped in a sterile bag, thus eliminating the concept of contactless. In November 2010, the Microsoft Kinect (MK) 3D depth camera system (Microsoft Corp) was launched as a device for the Xbox 360 games console. The first descriptions of MK for medical use were in relation to physical and cognitive rehabilitation [37]. Subsequent experiences in this field showed that additional studies were required on issues such as effectiveness, commitment, and usability [38-40]. Its use in an operating room was first reported in 2011, at Sunnybrook Hospital in Toronto, when it was used to view magnetic resonance imaging and computed tomography scans, eventually giving rise to the GestSure system [13]. In 2012, the Leap Motion Controller (LMC; Leap Motion Inc) was launched, and in July 2013, the Myo armband (Thalmic Labs) was launched.

Construct validity [41,42], concurrent validity [43,44], and predictive validity [45,46] studies, as well as systematic reviews [47,48], have shown that simulation in virtual reality environments is an effective tool for motor skills learning in MIS. However, the high cost of virtual reality and augmented reality simulators calls for the development of new, portable low-cost solutions enabling ubiquitous learning. New COTS technologies that allow hand gestures and instrument movements to be detected open up an interesting field of exploration for the development and validation of new simulation models in virtual environments. One of the objectives of this systematic review was to recognize the existence of developments in this area.

### Objectives

The aim of this systematic review was to provide an account of the state of the art of COTS devices in the detection of manual gestures in surgery and to identify their use as a simulation tool for motor skills teaching in MIS.

## Methods

### Article Retrieval

A search was conducted in the electronic databases PubMed, Excerpta Medica database (EMBASE), ScienceDirect, Espacenet, OpenGrey, and the Institute of Electrical and Electronics Engineers (IEEE) for articles published between January 2000 and December 2017, using combinations of the following Medical Subject Headings (MeSH) terms: *surgery*, *computer simulation*, *simulation training*, *laparoscopy*, *minimally invasive surgical procedures*, *robotic surgical procedures*, and *virtual reality*. The following were used as free terms: *commercial off-the-shelf*, *COTS*, *surgical education*, *surgical simulation*, *Wii*, *Microsoft Kinect*, *Xbox Kinect*, *Leap Motion*, *Leap Motion Controller*, *Myo armband*, and *gesture control*. The search strategy used a combination of MeSH terms and free terms. Boolean operators (AND and OR) were used to expand, exclude, or join keywords in the search. The devised strategy was applied first to PubMed and then to the remaining databases.

The search was limited to English-language publications and was complemented using the snowballing technique to identify relevant articles in the references of articles returned by our search [49]. A manual search was also conducted on the indices of the following publications: *Surgical Endoscopy*, *Surgical Innovation*, *Minimally Invasive Therapy and Allied Technologies*, the *Journal of Medical Internet Research*, and the *Journal of Surgical Education*. The snowballing search and the manual reviews enabled the retrieval of conference proceedings, letters to the editor, and simple concept descriptions. A MeaSurement Tool to Assess systematic Reviews (AMSTAR) [50] and Preferred Reporting Items for Systematic Reviews and Meta-Analysis (PRISMA) [51] checklists were used to ensure the quality of the review. In total, 3 authors assessed the risk of bias. Disagreement on bias assessment and the interpretation of results was resolved by consensus discussions.

### Study Selection

**Figure 1:**
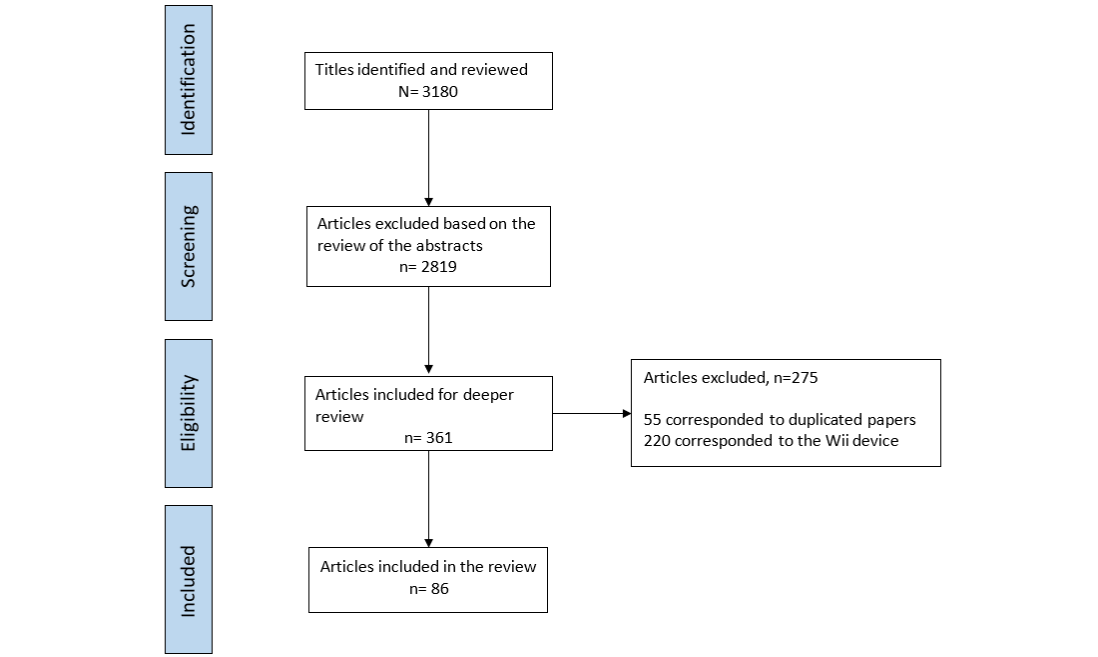
Flow diagram of studies through the review.

A total of 3180 studies were identified, and the abstracts were reviewed to determine whether they met the inclusion and exclusion criteria. The inclusion criteria were (1) original research articles, (2) proof-of-concept or prototype user testing and feasibility testing studies, (3) studies conducted in surgical environments (preoperative, intraoperative, or postoperative), and (4) studies carried out in real or simulated surgical settings. The exclusion criteria were (1) studies on COTS devices requiring hand contact, (2) studies conducted in nonsurgical clinical environments, and (3) studies on the technical description of devices that did not include criteria of clinical usability, feasibility, or acceptance as an outcome. Studies on COTS devices requiring hand contact (ie, Wii) were excluded from the analysis. After the first review of the titles and abstracts, 361 studies were selected, 220 of which corresponded to the Wii device and were therefore discarded. Of the 141 remaining articles, 55 were duplicate references. After reading the full texts of these studies, 86 were deemed to have met the search selection criteria. The search and selection processes are summarized in Figure 1.

We used a standardized form for data extraction, which included the following items: study, device on which the study was conducted, year of publication, aim, type of study, intervention, metrics, sample, and results and conclusions; clinical areas in which the study was conducted and types of surgical intervention (Tables 1-4) (see Multimedia Appendices 1-3 for the full Tables 1-3) and use of gesture-based COTS devices in surgery (Table 5). In total, 2 authors (FAL and MM) screened all the articles individually. Discrepancies were always resolved through discussion with the senior author (FSR) whenever necessary. All the data were analyzed qualitatively and quantitatively.

## Results

Of the 86 articles identified, 43 (50%) were on MK, 31 (36%) were on the LMC, 2 compared MK with the LMC [77,113], 1 compared the LMC with the Myo armband [58], 1 compared MK with the LMC and the Myo armband [52], 6 were on web, video, or commercial cameras (7%), and 2 reviewed gesture interaction in general [59,65]. The data and detailed information on the studies reviewed are shown in Tables 1-3 (see Multimedia Appendices 1-3 for the full Tables 1-3). The results are organized by the type of COTS device used (Tables 1-3, see Multimedia Appendices 1-3 for the full Tables 1-3), by the type of surgical specialties in which COTS devices were used (Table 4), and by the type of use made of COTS devices in surgery, including simulation for motor skills learning (Table 5).

**Table 1:** Summary of included studies evaluating Microsoft Kinect.

Study	Aim	Type of study	Intervention	Sample	Results/Conclusions
[17]	To describe a system for the interactive exploration of medical images through a gesture-controlled interface using MK^a^.	Proof-of-concept.	Manipulation of CT^b^, MRI^c^ and Positron emission tomography images.	Not described.	As the interface does not require direct contact or calibration, it is suitable for use in the operating room.
[99]	To explore the potential simplifications derived from using 3D^d^ sensors in medical augmented reality applications by designing a low-cost system.	Proof-of-concept.	Augmented reality in Medicine.	Not described.	The concept is feasible but the whole process is still too time-consuming to be executed in real time.
[101]	To present an augmented reality magic mirror for anatomy teaching.	Proof-of-concept.	Augmented reality in Medicine. Anatomy education.	A hospital and a school.	The system can be used for educational purposes, to improve communication between doctor and patients. A possible use for anatomy teaching in surgery is not mentioned.
[5]	To evaluate the response time and usability (gestures and voice commands) compared with mouse and keyboard controls.	Prototype user testing and feasibility testing.	Manipulation of CT images.	2 radiologists and 8 forensic pathologists who recreated 12 images.	Users took 1.4 times longer to recreate an image with gesture control and rated the system 3.4 out of 5 for ease of use in comparison with the keyboard and mouse. The voice recognition system did not work properly.
[84]	To develop a system to allow the surgeon to interact with the standard PACS system during sterile surgical management of orthopedic patients.	Proof-of-concept.	Manipulation of radiological images in orthopedics.	Not described.	This is the first example of this technology being used to control digital X-rays in clinical practice.
[83]	To present a sterile method for the surgeon to manipulate images using touchless freehand gestures.	Experiment.	Manipulation of MRI images.	9 veterinary surgeons. 22 students.	The hypothesis that contextual information integrated with hand trajectory gesture information can significantly improve the overall recognition system performance was validated. The recognition accuracy was 98.7%
[76]	To evaluate an MK-based interaction system for manipulating imaging data using ‘Magic Lens visualization.‘	Proof-of-concept in the operating room.	Manipulation of radiological images.	A laryngoplasty.	The surgeon can manipulate the preoperative information with the intraoperative video and the simulations to correctly place the implant.
[79]	To compare the accuracy and speed of interaction of MK with that of a mouse. To study the performance of the interaction methods in rotation tasks and localization of internal structures in a 3D dataset.	User testing.	Manipulation of radiological images.	15 users.	The gesture-based interface outperformed the traditional mouse with respect to time and accuracy in the orientation and rotation task. The mouse was superior in terms of accuracy of localization of internal structures. However, the gesture-based interface was found to have the fastest target localization time.
[74]	To develop a user-friendly touchless system for controlling the presentation of medical images based on hand gesture recognition in the operating room.	Proof-of-concept in the operating room.	Manipulation of radiological images in orthopedic surgery.	Not described.	The system does not require calibration and was adapted to the surgical environment following the principles of asepsis/antisepsis.
[30]	To present a touchless gesture interface that allows the surgeon to control medical images using hand gestures.	Proof-of-concept and prototype feasibility testing.	Manipulation of CT images.	Enucleation of 4 tumors in 3 urology patients.	First description in the literature of a gesture user interface using MK in the operating room in in-vivo surgery, showing that it is an efficient and low-cost solution.
[100]	To develop a low-cost augmented reality interface projected onto a mannequin simulator.	Proof-of-concept.	Augmented reality for education in Medicine.	A physical simulator, video projector, Wii Remote and MK.	The manipulations obtained using MK were similar to those described with the Wii.
[67]	To develop a version of a gesture-based system for controlling images.	Proof-of-concept.	Manipulation of MRI images.	Resection of a glioma.	Except for the scanning movement, each movement was recognized with great accuracy. The algorithm can be installed in the clinical area.
[128]	To use MK to operate an automated operating-room light system.	Prototype user testing.	Manipulation of operating room lights.	18 volunteers.	The gestures were easy to learn and the movement of the light beam was sufficiently precise.
[102]	To create a touchless head tracking system for an immersive virtual operating room.	Proof-of-concept.	Virtual reality for simulation and education in surgery.	A 3D virtual operating room with a virtual operating table.	Using MK, it was possible to implement a very accurate interactive tracking system regardless of the complexity of the virtual reality system.
[85]	To present a new prototype that allows the user to control the OsiriX system with finger gestures using a low-cost depth camera.	Proof-of-concept and prototype feasibility testing.	Manipulation of CT images.	4 forensic pathologists, 1 radiologist and 1 engineer.	On average, 4.5 min were required to learn to use the system. Participants rated the intuitiveness of the gestures with 3.8 out of 5 and control of the images with 3.8 out of 5. The low cost of the system makes it affordable for any potential user.
[104]	To present a new immersive surgical training system.	Proof-of-concept and prototype fidelity testing.	Virtual reality for education in surgery.	Cholecystectomy training on animal tissue blocks.	Initial feedback from the residents showed that the system is much more effective than the conventional videotaped system.
[60]	To test a speech and gesture-controlled interventional radiology system.	User testing.	Manipulation of CT and angiography images.	10 radiology residents used commands under different lighting conditions during 18 angiographies and 10 CT- guided punctures.	93% of commands were recognized successfully. Speech commands were less prone to errors than gesture commands. 60% of participants would use the application in their routine clinical practice.
[86]	To develop an image operation system for image manipulation using a motion sensor.	Proof-of-concept.	Manipulation of angiographic images.	Not described.	The system can be implemented as a useful tool in angiography for controlling image viewing using gestures in the operating room.
[19]	The working hypothesis is that contextual information such as the focus of attention, integrated with gestural information, can significantly improve overall system recognition performance compared with interfaces relying on gesture recognition alone.	Ethnographic study. Experiment. Survey.	Manipulation of MRI images.	10 veterinary surgeons. 20 volunteers.	The surgeon’s intention to perform a gesture can be accurately recognized by observing environmental cues (context). The hypothesis was validated by a drop in the false positive rate of gesture recognition from 20.76% to 2.33%. A significant rate of reduction of the mean task completion time indicated that the user operates the interface more efficiently with experience. The tracking algorithm occasionally failed in the presence of several people in the camera’s field of view.
[96]	To examine the functionality and usability of MK to complete the visualization of 3D anatomical images.	User testing. Survey.	Manipulation of anatomical images.	32 participants: Medical students, professors and anatomy laboratory staff.	MK users reached accuracy levels almost identical to those who used a mouse, and spent less time on performing the same tasks. MK showed potential as a device for interaction with medical images.
[103]	To examine usability for navigating through 3D medical images using MK compared with a traditional mouse.	User testing. Survey.	Manipulation of anatomical images. Education.	17 veterinary students.	Improvements should be made to MK before it can be implemented as a device for medical use. The preferred method was the mouse. MK has the potential to reduce time on the task.
[13]	To develop a prototype and to examine the feasibility of this new device to help bridge the sterility barrier and eliminate the time and space gap that exists between image review and visual correlation with real-time operative field anatomy.	Proof-of-concept and prototype feasibility testing.	Manipulation of CT and MRI images.	2 MIS^e^ procedures and 4 open procedures performed by a surgeon.	The system worked well in a wide range of lighting conditions and procedures. There was an increase in the use of intraoperative image consultation. The gesture library was intuitive and easy to learn. Gestures were mastered within 10 min.
[61]	To investigate a solution for manipulating medical images using MK.	Proof-of-concept and prototype feasibility testing.	Manipulation of CT images.	29 radiologists (diagnostic and interventional).	The potential of the device to enhance image-guided treatment in an interventional radiology suite while maintaining a sterile surgical field was demonstrated. 69% of those surveyed believed that the device could be useful in the interventional radiology field.
[112]	To investigate the need for posture and position training during bronchoscopy using a tool called ETrack	Pilot study.	Analysis of the operator’s movements during a bronchoscopy. Education.	Not described.	The results highlight the importance of posture during bronchoscopy and the need to implement a training module for the simulator.
[71]	To evaluate a new touchless, portable, low-cost 3D measurement system for objective breast assessment.	Concurrent validation study.	Calculation of breast implant volumes.	9 silicone implants of known volumes.	The implant volumes were calculated with an error margin of 10%. Reproducibility was satisfactory. The system was validated for clinical use.
[106]	To describe a gesture-controlled 3D teaching tool in which temporal bone anatomy is manipulated without using a mouse or keyboard. To provide a teaching tool for patient-specific anatomy.	Proof-of-concept.	Manipulation of anatomical images. Education.	0.15 mm slice thickness cadaveric temporal bone images.	The interactive 3D model developed seems promising as an educational tool.
[62]	To develop hand recognition software based on MK, linked to an interventional CT, to manipulate images.	Feasibility testing	Manipulation of CT images in surgery.	10 interventional radiology procedures. 1 operator.	Tested on 10 procedures, feasibility was 100%. The system also allowed information to be obtained without using the CT system interface or a third party, and without the loss of operator sterility.
[131]	To present a novel method for training intentional and nonintentional gesture recognition.	Experiment.	Performance of a simulated brain biopsy on a mannequin assisted by images manipulated using gestures.	19 subjects.	Continuous gesture recognition was successful 92.26% of the time with a reliability of 89.97%. Significant improvements in task completion time were obtained through the context integration effect.
[113]	To evaluate 2 contactless hand tracking systems, the LMC^f^ and MK, for their potential to control surgical robots.	Experiment.	Manipulation of robots in surgery.	4 trained surgeons.	Neither system has the high level of accuracy and robustness that would be required for controlling medical robots.
[107]	To use a projector for visualization and to provide intuitive means for direct interaction with the information projected onto the surgical surface, using MK to capture the interaction zone and the surgeon’s actions on a deformable surface.	Proof-of-concept.	Augmented reality in surgery.	Not described.	The system eliminates the need for the surgeon to look at a location other than the surgical field. It therefore removes distractions and enhances his or her performance. It not only provides the surgeon with medical data during the intervention, but also allows interaction with such information by using gestures.
[10]	To present an ethnographic study of a system based on MK developed to allow touchless control of medical images during vascular surgery. The study aims to go beyond demonstrating technical feasibility in order to understand the collaborative practices that emerge from its use in this context.	Ethnographic study.	Manipulation of radiological images.	Endovascular suite of a large hospital.	With touchless interaction, the visual resources were embedded and made meaningful in the collaborative practices of surgery. The importance of direct and dynamic control of the images by the clinicians in the context of talks and in the context of other artefact use is discussed.
[130]	To evaluate a system for manipulating an operating table using gestures.	Prototype user testing.	Manipulation of an operating table.	15 participants.	Major problems were encountered during gesture recognition and with obstruction by other people in the interaction area due to the size and layout of the operating room. The system cannot yet be integrated into a surgical environment.
[110]	To study the technical skills of colonoscopists using MK for motion analysis to develop a tool to guide colonoscopy education and to select discriminative motion patterns.	Construct validity study.	Analysis of the movements of the operator during a colonoscopy.	10 experienced and 11 novice endoscopists.	Certain types of metric can be used to discriminate between experienced and novice operators.
[72]	To develop a 3D surface imaging system and to assess the accuracy and repeatability on a female mannequin.	Interrater reliability study.	Measurement of the surface distances of the breast on a mannequin.	A female mannequin.	MK seems to be a useful and feasible system for capturing 3D images of the breast. There was agreement between the measurements obtained by the system and those taken manually with a measuring tape.
[105]	To present a new surgical training system.	Proof-of-concept.	Real-time immersive 3D surgical training. Education.	Not described.	Preliminary experiments show that this immersive training system is portable, effective and reliable.
[68]	To present the development and clinical testing of a device that enables intraoperative control of images with hand gestures during neurosurgical procedures.	Proof-of-concept. Initial clinical testing.	Manipulation of MRI images.	30 neurosurgical operations.	OPECT demonstrated high effectiveness, simplicity of use and precise recognition of the individual user profile. In all cases, surgeons were satisfied with the performance of the device.
[68]	To test whether an automatic motion analysis system could be used to explore if there is a correlation in scope movements and the level of experience of the surgeon performing the bronchoscopy.	Construct validity study. Prospective, comparative study.	Analysis of the operator’s movements during a bronchoscopy. Education.	11 novice, 9 intermediate and 9 experienced bronchoscopy operators performed 3 procedures each on a bronchoscopy simulator.	The motion analysis system could discriminate between different levels of experience. Automatic feedback on correct movements during self-directed training on simulators might help new bronchoscopists learn how to handle the bronchoscope like an expert.
[77]	To compare 2 commercial motion sensors (MK and the LMC) to manipulate CT images, in terms of their utility, usability, speed, accuracy and user acceptance.	Two-strand sequential observational study. Qualitative and quantitative descriptive field study using a semi-structured questionnaire.	Manipulation of CT images.	42 participants: radiologists, surgeons and interventional radiologists.	Marginal to average acceptability of the 2 devices. MK was found to be more useful and easier to use, but the LMC was more accurate. Further research is required to establish the design specifications, installation guidelines and user training requirements to ensure successful implementation in clinical areas.
[57]	To develop an integrated and comprehensive operating room information system compatible with HL7 and DICOM (MediNav). A natural user interface is designed specifically for operating rooms based on MK.	Prototype user testing.	Users tested the application’s various modules.	A prototype system is tested in a live operating room at an Iranian teaching hospital. 30 general surgeries.	The results of usability tests are promising, and indicate that integration of these systems into a complete solution is the key. Touchless natural user interfaces can help to collect and visualize medical information in a comprehensive manner.
[75]	To propose a novel system to visualize a surgical scene in augmented reality using the different sources of information provided by a C-arm and MK.	Prototype user testing.	Augmented reality in orthopedic surgery.	Simulations of 12 orthopedic procedures. 5 participating clinicians, 3 experienced surgeons, 2 fourth-year medical students.	The system showed promising results with respect to better surgical scene understanding and improved depth perception using augmented reality in simulated orthopedic surgery.
[114]	To explore 3D perception technologies in the operating room.	Ethnographic. Prototype testing.	Detection of the interaction between operating staff and the robot.	Not described.	The paper described a supervision system for the operating room that enables intention tracking. The system had low latency, good registration accuracy and high tracking reliability, which make it useful for workflow monitoring, tracking and avoiding collisions between medical robots and operating room staff.
[125]	To use MK and color markers to track the position of MIS instruments in real time.	Comparative study between MK and the SinaSim trainer.	Movement of the instrument to position its tip in 81 holes of a Plexiglas plate on 5 occasions.	1 user.	Although the new method had inferior accuracy compared with mechanical sensors, its low cost and portability make it a candidate for replacing traditional tracking methods.
[80]	To compare 3 different interaction modes for image manipulation in a surgery setting: 1) A gesture-controlled approach using MK; 2) verbal instructions to a third party; and 3) direct manipulation using a mouse.	Crossover randomized controlled trial with blocked randomization.	Interaction modes were direct manipulation using a mouse, verbal instructions given to a third party, and gesture-controlled manipulation using MK.	30 physicians and senior medical students	Under the premise that a mouse cannot be used directly during surgery, gesture-controlled approaches were shown to be superior to verbal instructions for image manipulation.
[121]	To evaluate the feasibility, validity, and reliability of the training system for motion parameter and ergonomic analyses between different experience levels of surgeons using the NDI Polaris System and MK camera.	Construct validity, concurrent validity and test-retest reliability. Prospective blinded study.	Tying of intra-corporeal MIS knots.	10 MIS novices, 10 intermediate level and 10 experts.	Validity and reliability of the self-developed sensor and expert model-based MIS training system ‘iSurgeon’ were established.
[73]	To analyze preoperative breast volume in patients with breast cancer in order to predict implant size for reconstruction.	Exploratory study.	MK was used to acquire 3D images of the patients’ breasts before surgery and after surgery.	10 patients.	This study showed the feasibility of using fast, simple and inexpensive 3D imaging technology for predicting implant size before surgery, although there were significant technical challenges in determining breast volume by surface imaging.
[52]	To evaluate the feasibility of using 3 different gesture control sensors (MK, the LMC and the Myo armband) to interact in a sterile manner with preoperative data as well as in settings of an integrated operating room during MIS.	Pilot user study.	2 hepatectomies and 2 partial nephrectomies on an experimental porcine model.	3 surgeons.	Natural user interfaces are feasible for directly interacting, in a more intuitive and sterile manner, with preoperative images and integrated operating room functionalities during MIS. The combination of the Myo armband and voice commands provided the most intuitive and accurate natural user interface.

^a^MK: Microsoft Kinect.

^b^CT: Computed Tomography.

^c^MRI: magnetic resonance imaging.

^d^3D: 3-dimensional.

^e^MIS: minimally invasive surgery.

^f^LMC: Leap Motion Controller.

**Table 2:** Summary of included studies evaluating the Leap Motion Controller.

Study	Aim	Type of study	Intervention	Sample	Results/Conclusions
[63]	To evaluate the implementation of a low-cost device for touchless PACS control in an interventional radiology suite. To demonstrate that interaction with gestures can decrease the duration of the procedures, the risk of re-intervention, and improve technical performance.	Proof-of-concept and prototype feasibility testing.	Manipulation of images in interventional radiology.	Interventional radiology suite.	The LMC^a^ is a feasible, portable and low-cost alternative to other touchless PACS interaction systems. A decrease in the need for re-intervention was reported, but no explanation was given of how it was measured.
[54]	To present the first experience of using new systems for image control in the operating room: the LMC and OsiriX.	Proof-of-concept.	Manipulation of CT^b^ and MRI^c^ images.	2 general surgeons, 1 urologist, 3 orthopedic surgeons and 2 surgeons	The average training time was 5 min. The system is very cost-effective, efficient and prevents contamination during surgery. First experience of using the LMC to control CT and MRI images during surgery.
[116]	To validate the possibility of performing precise telesurgical tasks by means of the LMC.	Comparative study of the Sigma.7 electro-mechanical device and the LMC.	Peg transferring task and answering a questionnaire. The success rate of peg transfers.	10 researchers.	The results allowed the authors to confirm that fine tracking of the hand could be performed with the LMC. The observed performance of the optical interface proved to be comparable with that of traditional electro-mechanical devices.
[87]	To describe a piece of software for image processing with OsiriX using finger gestures.	Proof-of-concept.	Manipulation of radiological images.	Not described.	It is possible to implement gesture control of medical devices with low-cost, minimal resources. The device is very sensitive to surface dirt and this affects performance. The device favors the occlusion phenomenon.
[113]	To evaluate 2 contactless hand tracking systems, the LMC and MK^d^, for their potential to control surgical robots.	Experiment.	Manipulation of robots in surgery.	4 trained surgeons.	Neither system has the high level of accuracy and robustness that would be required for controlling medical robots.
[129]	To evaluate the LMC for simple 2-dimensional interaction and the action of entering a value.	Proof-of-concept and prototype testing.	Manipulation of medical information and operating room lights.	A 90-min conference on computer science and untrained users.	The user cases should be carefully classified and the most appropriate gestures for each application should be detected and implemented. Optimal lighting conditions for the LMC have still not been evaluated as unwanted light with deterioration of the IR light emitted may lead to a reduction in the recognition rate.
[81]	To compare the average time required by the conventional method using a mouse and an operating method with a finger-motion sensor.	Observational study.	Manipulation of angiographic images.	11 radiologists who observed a simulated clinical case.	After a practice time of 30 min, the average operation time by the finger method was significantly shorter than that by the mouse method.
[14]	To develop a workstation that allows intraoperative touchless control of diagnostic and surgical images in dentistry.	Prototype user testing.	Manipulation of radiological images.	2 surgeons. A case series of 11 dental surgery procedures.	The system performed very well. Its low cost favors its incorporation into clinical facilities of developing countries, reducing the number of staff required in operating rooms.
[88]	To propose an interface to control hand gestures and gestures with hand-held tools. In this approach, hand-held tools can become gesture devices that the user can use to control the images.	Prototype user testing.	Manipulation of ultrasound images.	12 participants.	Users were able to significantly improve their performance with practice.
[56]	To develop a software application for the manipulation of a 3D^e^ pancreatic or liver tumor model by using CT and real-time elastography data.	Proof-of-concept.	Manipulation of CT and real-time elastography images.	15 patients with liver cancer and 10 patients with pancreatic cancer.	A 3D model of liver and pancreatic tumors was successfully implemented with a hands-free interaction device suitable for sterile environments and for aiding diagnostic or therapeutic interventions.
[117]	To present a new gesture recognition system for manipulating 2 surgical robots in a virtual simulator.	Proof-of-concept.	Manipulation of robots in surgery.	2 surgical robots in a virtual simulator.	The device provided satisfactory accuracy and speed. It requires a more complete Application Programming Interface.
[90]	To propose a web-based interface to retrieve medical images using gestures.	User testing. Pilot study.	Manipulation of radiological images.	2 users.	User feedback was positive. Users reported fatigue with prolonged use of gestures. Additional studies are required to validate the interface.
[64]	To describe the use of the LMC for image manipulation during hepatic transarterial chemoembolization and internal radiotherapy procedures.	Proof-of-concept.	Manipulation of images in interventional radiology.	Not described.	Gesture-based imaging control may lead to increased efficacy and safety with decreased radiation exposure during hepatic transarterial chemoembolization procedures.
[77]	To compare 2 commercial motion sensors (MK and the LMC) to manipulate CT images, in terms of their utility, usability, speed, accuracy and user acceptance.	Two-strand sequential observational study. Qualitative and quantitative descriptive field study using a semi-structured questionnaire.	Manipulation of CT images.	42 participants: radiologists, surgeons and interventional radiologists.	Marginal to average acceptability of the 2 devices. MK was found to be more useful and easier to use, but the LMC was more accurate. Further research is required to establish the design specifications, installation guidelines and user training requirements to ensure successful implementation in clinical areas.
[91]	To evaluate a new method for image manipulation using a motion sensor.	Observational study. User testing and proof-of-concept.	Manipulation of radiological images in dentistry.	14 students. 6 images.	Using the system, several processes can be performed quickly with finger movements. Using gestures was significantly superior to using a mouse in terms of time.
[92]	To develop a new system for manipulating images using a motion sensor.	Observational study.	Manipulation of radiological images in dentistry.	14 students. 25 images.	The operation time with the LMC was significantly shorter than with the conventional method using a mouse.
[108]	To design a virtual 3D online environment for motor skills learning in MIS^f^ using exercises from the MISR-VR. The environment is designed in Unity, and the LMC is used as the device for interaction with the MIS forceps.	Letter to the editor.	None.	Not described	If it can be shown that 3D online environments mediated by natural user interfaces enable motor skills learning in MIS, a new field of research and development in the area of surgical simulation will be opened up.
[124]	Patent for accurate 3D instrument positioning.	Patent.	None.	Not described	Representing, on an output display, 3D positions and orientations of an instrument while medical procedures are being performed.
[69]	To describe the configuration for using the LMC in neurosurgery for image manipulation during a surgical procedure.	User testing.	Manipulation of images during a surgical procedure.	Resection of a meningioma and sarcoma surgery.	The learning curve only took 30 min. Although the main disadvantage was the lack of standardization of the gestures, the LMC is a low-cost, reliable and easily personalized device for controlling images in the surgical environment.
[109]	To develop skills in students and professionals using computer simulation technologies based on hand gesture capture systems.	User testing.	Description of the virtual environment.	Not described.	Simulation and new gesture recognition technologies open up new possibilities for the generation of computer-mediated procedures for medical training.
[93]	To present a gesture-controlled projection display that enables a direct and natural physician-machine interaction during CT-based interventions.	User testing (pilot and main).	8 tasks manipulating CT images.	12 participants (biomedical engineers, medical students and radiologists).	Gesture recognition is robust, although there is potential for improvement. The gesture training times are less than 10 min, but vary considerably between study participants.
[94]	To develop an anatomy learning system using the LMC.	User testing.	Manipulation of 220 anatomical images.	30 students and lecturers from an anatomy department.	The anatomy learning system using the LMC was successfully developed and it is suitable and acceptable as a support tool in an anatomy learning system.
[123]	To study the possibility of tracking laparoscopic instruments using the LMC in a box trainer.	Experiment.	3 static experiments and 1 dynamic experiment.	1 user.	The LMC had acceptable precision for tracking laparoscopic instruments in a box trainer.
[126]	To assess the potential of the LMC to track the movement of hands using MIS instruments.	Construct validity, concurrent validity. Comparative study with the InsTrac.	Passing a thread through pegs using the eoSim simulator.	3 experts and 10 novices.	The LMC is able to track the movement of hands using instruments in a MIS box simulator. Construct validity was demonstrated. Concurrent validity was only demonstrated for time and instrument path distance. A number of limitations to the tracking method used by LMC have been identified.
[118]	To explore the use of the LMC in endonasal pituitary surgery and to compare it with the Phantom Omni.	Comparative study between the LMC and the Phantom Omni.	16 resections of simulated pituitary gland tumors using a robot manipulated by the Phantom Omni and by the LMC.	3 neurosurgeons.	Users were able to achieve a very similar percentage of resection and procedure duration using the LMC.
[95]	To try to interact with medical images via a web browser using the LMC.	Prototype user testing.	Rotation, panning, scaling and selection of slices of a reconstructed 3D model based on CT or MRI.	1 user.	It is feasible to build this system and interaction can be carried out in real time.
[58]	To analyze the value of 2 gesture input modalities (the Myo armband and the LMC) versus 2 clinically established methods (task delegation and joystick control).	User study. Comparative study.	Simulating a diagnostic neuroradiological vascular treatment with 2 frequently used interaction tasks in an experimental operating room.	10 neuroradiologists	Novel input modalities have the potential to carry out single tasks more efficiently than clinically established methods.
[120]	To investigate the potential of a virtual reality simulator for the assessment of basic laparoscopic skills, based on the LMC	Face and construct validity.	3 basic tasks: camera navigation, instrument navigation, and two-handed operation.	2 groups of surgeons (28 experts and 21 novices).	This study provides evidence of the potential use of the LMC for assessing basic laparoscopic skills. The proposed system allows the dexterity of hand movements to be evaluated.
[52]	To evaluate the feasibility of using 3 different gesture control sensors (MK, the LMC and the Myo armband) to interact in a sterile manner with preoperative data as well as in settings of an integrated operating room during MIS.	Pilot user study.	2 hepatectomies and 2 partial nephrectomies on an experimental porcine model.	3 surgeons	Natural user interfaces are feasible for directly interacting, in a more intuitive and sterile manner, with preoperative images and integrated operating room functionalities during MIS. The combination of the Myo armband and voice commands provided the most intuitive and accurate natural user interface.
[127]	To evaluate the LMC as a tool for the objective measurement and assessment of surgical dexterity among users at different experience levels.	Construct validity study.	Surgical knot tying and manual transfer of objects.	11 participants.	The study showed 100% accuracy in discriminating between expert and novice performances.
[66]	To design an affordable and easily accessible endoscopic third ventriculostomy simulator based on the LMC, and to compare it with the NeuroTouch for its usability and training effectiveness.	Concurrent and construct validity study.	4 ellipsoid practice targeting tasks and 36 ventricle targeting tasks.	16 novice users and 2 expert neurosurgeons	An easy-access simulator was created, which has the potential to become a training tool and a surgical training assessment tool. This system can be used for planning procedures using patient datasets.
[119]	To present the LMC as a novel control device to manipulate the RAVEN-II robot.	Comparative study between the LMC and the electro-mechanical Sigma.7.	Comparison of peg manipulations during a training task with a contact-based device (Sigma.7).	3 operators.	With contactless control, manipulability is not as good as it is with contact-based control. Complete control of the surgical instruments is feasible. This work is promising for the development of future human-machine interfaces dedicated to robotic surgical training systems.
[98]	To evaluate the effect of using virtual reality surgery on the self-confidence and knowledge of surgical residents (the LMC and Oculus Rift).	Multisite, single-blind, parallel, randomized controlled trial.	The study group used the virtual reality surgery application. The control group used similar content in a standard presentation.	95 residents from 7 dental schools.	Immersive virtual reality experiences improve the knowledge and self-confidence of the surgical residents.
[97]	To develop and validate a novel training tool for Le Fort I osteotomy based on immersive virtual reality (the LMC and Oculus Rift).	Face and content validity.	A pre-intervention questionnaire to understand training needs and a postintervention feedback questionnaire.	7 consultant oral and maxillofacial surgeons.	The results confirmed the clinical applicability of virtual reality for delivering training in orthognathic surgery.
[70]	To investigate the feasibility and practicability of a low-cost multimodal head-mounted display system in neuroendoscopic surgery (the LMC and Oculus Rift).	Proof-of-concept in the operating room.	Ventriculocysto- cisternostomy. Ventriculostomy. Tumoral biopsy.	21 patients with ventricular diseases. 1 neurosurgeon.	The head-mounted display system is feasible, practical, helpful, and relatively cost efficient in neuroendoscopic surgery.

^a^LMC: Leap Motion Controller.

^b^CT: Computed Tomography.

^c^MRI: magnetic resonance imaging.

^d^3D: 3-dimensional.

^e^MK: Microsoft Kinect.

^f^MIS: minimally invasive surgery.

**Table 3:** Summary of included studies evaluating other devices.

Study	Device	Aim	Type of study	Intervention	Results/Conclusions
[53]	Camera with Complementary Metal-Oxide-Semiconductor sensor	To propose an architecture for a real-time multimodal system to provide a touchless user interface in surgery.	Prototype user testing.	Gesture detection in computer-assisted surgery.	The preliminary results show good usability and rapid learning. The average time to click anywhere on the screen was less than 5 seconds. Lighting conditions affected the performance of the system. The surgeon showed strong interest in the system and satisfactorily assessed the use of gestures within the operating room.
[82]	Webcam	To describe a vision-based system that can interpret gestures in real time to manipulate objects within a medical data visualization environment.	Prototype user testing.	Manipulation of medical data (radiology images and selection of medical records) and movement of objects and windows on the screen.	The system implemented in a sterile environment demonstrated performance rates between 95% and 100%.
[27]	Canon VC-C4 color camera	To describe a vision-based gesture capture system that interprets gestures in real time to manipulate medical images.	Beta testing during a surgical procedure. Experiment.	A beta test of a system prototype was conducted during a live brain biopsy operation, where neurosurgeons were able to browse through MRI^a^ images of the patient’s brain using the sterile hand gesture interface.	Gesture recognition accuracy was 96%. For every repeat of trials, the task completion time decreased by 28% and the learning curve levelled off at the 10th attempt. The gestures were learned very quickly and there was a significant decrease in the number of excess gestures. Rotation accuracy was reasonable. The surgeons rated the system as easy to use, with a rapid response, and useful in the surgical environment.
[26]	Canon VC-C4 camera	To evaluate the Gestix system.	Prototype user testing.	Manipulation of MRI images during a neurosurgical biopsy.	The system setup time was 20 min. The surgeons found the Gestix system easy to use, with a rapid response, and easy to learn. The system does not require the use of wearable devices.
[59]	Interaction with gestures in general	Fieldwork focusing on work practices and interactions in an angiography suite and on understanding the collaborative work practices in terms of image production and use.	Ethnographic study of minimally invasive image-guided procedures within an interventional radiology department.	Manipulation of radiological images.	The paper discusses the implications of the findings in the work environment for touchless interaction technologies, and suggests that these will be of importance in considering new input techniques in other medical settings.
[115]	Commercial video camera	To describe the development of Gestonurse, a robotic system for surgical instruments.	Proof-of-concept.	Surgical instrumentation using a robot.	95% of gestures were recognized correctly. The system was only 0.83 seconds slower when compared with the performance of a human instrument handler.
[65]	Touchless interaction systems in general	To understand and use common practices in the surgical setting from a proxemics point of view to uncover implications for the design of touchless interaction systems. The aim is to think of touchlessness in terms of its spatial properties. What does spatial separation imply for the introduction of the touchless control of medical images?	Ethnographic study.	Field observations of work practices in neurosurgery.	Alternative ideas, such as multiple cameras, are the kind of solution that these findings suggest. Such reflections and considerations can be revealed through careful analysis of the spatial organization of activity and proxemics of particular interaction mechanisms. However, it is very important to study current practice in order to speculate about new systems, because they in turn may alter practice.
[122]	Webcam	To present a system for tracking the movement of MIS^b^ instruments based on an orthogonal webcam system installed in a physical simulator.	Experiment.	Recording the movements of the instrument within an imaginary cube.	The results showed a resolution of 0.616 mm on each axis of work, linearity and repeatability in motion tracking, as well as automatic detection of the 3D position of the tip of the surgical instruments with sufficient accuracy. The system is a low-cost and portable alternative to traditional instrument tracking devices.
[52]	MK, the LMC^c^, the Myo armband and voice control	To evaluate the feasibility of using 3 different gesture control sensors (MK, the LMC and the Myo armband) to interact in a sterile manner with preoperative data as well as in settings of an integrated operating room during MIS.	Pilot user study.	2 hepatectomies and 2 partial nephrectomies on an experimental porcine model.	Natural user interfaces are feasible for directly interacting, in a more intuitive and sterile manner, with preoperative images and integrated operating room functionalities during MIS. The combination of the Myo armband and voice commands provided the most intuitive and accurate natural user interface.
[58]	The Myo armband and the LMC	To analyze the value of 2 gesture input modalities (the Myo armband and the LMC) versus 2 clinically established methods (task delegation and joystick control).	User study. Comparative study.	Simulating a diagnostic neuroradiological vascular treatment with 2 frequently used interaction tasks in an experimental operating room.	Novel input modalities have the potential to carry out single tasks more efficiently than clinically established methods.

^a^MRI: magnetic resonance imaging.

^b^MIS: minimally invasive surgery.

^c^LMC: Leap Motion Controller.

**Table 4:** Clinical areas and types of surgical intervention in which gesture-based commercial off-the-shelf devices were used.

Clinical areas	Types of surgical intervention	Studies
General surgery (N=7)	Intraoperative image control, image-guided minimally invasive surgery (adrenalectomy, pancreatectomy, liver resection, a Whipple procedure, as well as liver and pancreatic cancer and renal carcinoma resection), open and laparoscopic bile duct surgery, cholecystectomy, and hepatectomy and nephrectomy in an animal model.	[13,52-57]
Interventional radiology and angiography (N=7)	Arterial dilatation with balloon and umbrella devices, hepatic arterial chemoembolization and selective internal radiation therapy, abdominal computed tomography, and interventional neuroradiology.	[58-64]
Neurosurgery (N=7)	Biopsies, resection of brain gliomas, resection of a meningioma, ventriculostomy, and intraoperative image control.	[26,65-70]
Plastic surgery (N=3)	Measurement of breast implant volumes and measurement of distances on the breast surface.	[71-73]
Orthopedics (N=3)	Intraoperative image control.	[55,74,75]
Ear, nose, and throat (N=1)	Laryngoplasty.	[76]
Urology (N=2)	Enucleation of renal tumors and intraoperative image control.	[30,54]

**Table 5:** Use of gesture-based commercial off-the-shelf devices in surgery.

Use		Studies
**Manipulation of images in interventional radiology environments or in the operating room (N=42)**		
	Image manipulation	[5,13,14,17,19,26,27,30,52,54,56,58-64,67-69,74,76-95]
**Education and training**		
	Virtual or augmented reality for educational or interventional purposes (N=16)	[75,94,96-109]
	Training in endoscopy (bronchoscopy and colonoscopy; N=3)	[110-112]
**Robotic surgery (N=7)**		
	Robotics in surgery and in surgical instrumentation	[113-119]
**Tracking of hand or instrument movements during open or minimally invasive surgery**		
	Instrument tracking in MIS^a^ (N=7)	[108,120-125]
	Tracking of hand movements during MIS (N=2)	[109,126]
	Tracking of hand movements during open surgical knot tying (N=1)	[127]
**Simulation for skills learning in MIS** **(N=4)**		
	Simulation for motor skills learning in MIS	[66,108,120]
	Using patient-specific 3-dimensional images during MIS in real patients or simulators, and presurgical warm-up	[52,66,70,108]
**Other uses**				
	Ethnographic studies (N=5)	[59,65,78,83,114]
	Measurement of breast implant volumes and measurement of distances on the breast surface (N=3)	[71-73]
	Manipulation of the operating table and lights (N=4)	[128-130]

^a^MIS: minimally invasive surgery.

### Aims, Types of Study, Metrics, Samples, Results and Conclusions

In 78% (67/86) of the articles, the aim was to develop, create, present, describe, propose, examine, or explore a COTS-based system for gesture recognition in surgery. Most of the articles [65] identified in this systematic review were proof-of-concept or prototype user testing and observational and feasibility testing studies (Tables 1-3, see Multimedia Appendices 1-3 for the full Tables 1-3). In the 5 ethnographic studies included, the aim was to identify interactions between the staff and gesture-based COTS systems in interventional radiology departments or in the operating room [19,59,65,78,114]. In 4 studies, the aim was to compare the performance of MK with that of a mouse [5,79,80,96]; in 1 study, it was to compare the performance of the LMC with that of a mouse [81]; and in 4 studies, it was to compare different COTS devices [52,58,77,113]. In 10 studies, the aim was to evaluate face validity [97,120], content validity [97], construct validity [66,110,111,120,121,126,127,132], or concurrent validity of the devices [66,71,121,126]. A total of 7 studies involved experiments [19,26,113,115,122,123,131] and there was 1 patent application for an LMC-based application [124] and 1 interrater reliability study [72]. In addition, 1 study was a quasi-experimental prospective, blinded study with test-retest reliability [121]. Only 2 randomized controlled trials were identified [80,98], and when a tool for assessing risk of bias in randomized trials [133] was applied to them, it was found to be low in both.

In total, 25 out of 86 (29%) articles failed to describe the metric used, whereas 23 out of 86 (27%) used time as the main one. Given the varied nature of the design of the studies, the remaining 38 articles described multiple metrics such as performance rates, percentage of gesture recognition, accuracy of gesture recognition and/or speed of transmission thereof, measures of volume or distance, and questionnaires or interviews. Similarly, the sample types and numbers were very dissimilar: 17.4% of the articles did not describe the sample type, and the remainder stated that the samples comprised medical or veterinary students or specialists in several radiological or surgical specialties (Table 4).

### Interventions

The most common intervention (42 studies) was image manipulation in general radiology, ultrasound imaging, interventional radiology, angiography, computed tomography, magnetic resonance imaging, and real-time elastography (in the operating room, in the operative dentistry setting, or in the interventional radiology suites; Tables 1-3; see Multimedia Appendices 1-3 for the full Tables 1-3). Table 5 shows other uses identified for gesture-based COTS devices in surgical environments.

### Use of Commercial Off-The-Shelf Devices as Simulation Tools for Motor Skills Teaching in Minimally Invasive Surgery

In the field of skills learning in MIS, in 2013, Pérez et al first described the tracking of laparoscopic instruments using webcams, with encouraging results [122]. From 2016, several authors proposed the interesting possibility of using COTS devices for tracking laparoscopic instruments. Such devices include both the LMC [108,121,123,124] and MK [125]. In 2017, a portable low-cost simulator using the LMC [120] for basic motor skills learning in MIS was described, and so too were a simulator for endoscopic third ventriculostomy learning [66] and a head-mounted display system using Oculus Rift and the LMC to guide neuroendoscopic surgery by manipulating 3D images [70]. Others used the approach of tracking hand movements during MIS training [109,126]. Only 1 study explored the use of the LMC to assess surgical dexterity in tying surgical knots in open surgery [127].

Furthermore, 1 study compared 3 natural user interfaces (MK, the LMC, and the Myo armband) in combination with voice control to perform 2 hepatectomies and 2 partial nephrectomies on an experimental porcine model [52]; similar to the studies by Wright [66] and Xu [70], this study used 3D reconstructions of preoperative images of the patient, which were manipulated by gestures during surgery. However, the application of gesture control technology in these cases is not for training purposes but for surgical assistance and planification.

## Discussion

### Principal Findings

Using commercial devices to detect manual gestures in surgery is a very topical issue, given the need to manipulate medical images and for real-time 3D reconstructions during procedures without breaking asepsis and antisepsis protocols. Early studies published on this possibility used COTS systems with webcams, Complementary Metal-Oxide-Semiconductor-sensor cameras, and commercial digital cameras [26,27,53,82]. These pioneering studies showed that contactless interaction with images and medical information in environments such as operating rooms was possible using low-cost devices.

In this systematic review, MK and the LMC were identified as the most widely used COTS systems. MK was rated as a useful tool for the manipulation of medical data in sterile environments, with a positive rate of acceptance in 85% (39/46) of the studies on it. The LMC had a positive rate of acceptance in 83% (29/35) of the studies on it. The Myo armband was used to manipulate interventional neuroradiology images [58]. In addition, in a comparative study of the Myo armband, MK, and the LMC, they were used to manipulate images while hepatectomies and partial nephrectomies were being performed on an animal model [52]. In both cases, the device was rated highly. The main positive characteristics identified for the devices were the following: there was no need for contact; they were low-cost and portable; there was no need for calibration at the time of use; the gesture learning curve was easy; and the gesture recognition rates were high.

### Performance of Individual Devices

MK [30] and the LMC [14,81,87,134,135] both use infrared cameras. The MK system is based on the time-of-flight principle [61], whereas the LMC is based on a sensor for infrared optical tracking with stereo vision accuracy. The MK depth sensor works at a distance between 0.8 m and 3.5 m, and the interface tracks the skeleton of the system operator. The wide range of distances at which the device recognizes gestures presents problems when using it in close interaction. The LMC detects the positions of fine objects such as finger tips or pen tips in a Cartesian plane. Its interaction zone is an inverted cone of approximately 0.23 m³ and the motion detection range fluctuates between 20 mm and 600 mm [91,129]. The manufacturer reports an accuracy of 0.01 mm for fingertip detection, although 1 study showed an accuracy of 0.7 mm, which is considered superior to that achieved using MK [134,136]. The dimensions of the MK device are 280 mm (width), 71 mm (depth), and 66 mm (height) and its weight is 556 g, whereas those of the LMC are 76 mm (width), 30 mm (depth), and 13 mm (height) and its weight is 45 g.

Only 5 of the 46 (11%) studies that evaluated MK identified disadvantages relating to a longer latency time, difficulty in recreating an image when compared with a keyboard or mouse [5], limited gesture recognition, interference between the movements of different people in small environments [85,89,130], and the users’ preference for a mouse in a comparative study [96]. Various studies have highlighted the inaccuracy of MK in detecting finger movements [5,17,85,137], and the system also requires the use of large format screens [14,24,54,85,90]. The system was taken off the market in October 2017.

With regard to the LMC, once the 6 studies on robotics had been discarded, 4 articles were identified that presented limitations derived from using the device (18%). These studies noted alterations in performance when there was dirt on the surface of the device, as well as the limited number of gestures recognized owing to the occlusion phenomenon [87], alterations caused by ambient lighting [129], fatigue in some users [90], and a lack of studies validating the device for medical use [77].

The Myo armband was launched in 2013. This wearable wireless device is able to record electromyography via 8 stainless steel dry surface electrodes. It has a 9-axis inertial measurement unit sensor, haptic feedback, and Bluetooth communication capability. The main disadvantage is its limited sampling frequency of 200 Hz [138-140]. In total, 2 studies on the Myo armband were identified. The first concluded that the combination of the Myo armband and voice commands provided the most intuitive and accurate natural user interface [141]. The second compared the Myo armband and LMC with traditional image manipulation methods in surgery and concluded that the new input modalities had the potential to become more efficient [58].

### Commercial Off-The-Shelf Devices in Robotic Surgery

Studies on the application of gesture-based COTS devices in robot-assisted surgery failed to demonstrate usefulness, owing to either the high cost of the robotic arm when using commercial cameras in surgical instrumentation [115] or, in the case of the LMC, the need for a more robust Application Programming Interface [116,117] and the lack of sufficient accuracy and robustness for manipulating a medical robot [113]. However, an ethnographic study found that MK was useful for workflow monitoring and for avoiding collisions between medical robots and operating room staff [114]. A simulation study of endonasal pituitary surgery comparing the LMC with the Phantom Omni showed that surgeons achieved a very similar percentage of tumor mass resection and procedure duration using the LMC to control the robot [118]. Another study found that the robotic tools could be controlled by gestures for training purposes but that the level of control had yet to reach that of a contact-based robotic controller [119].

### Commercial Off-The-Shelf Devices in Training and Simulation

Studies on the use of COTS devices for gesture-based interfaces using the hand in the field of education in surgery refer to the use of virtual reality and augmented reality for teaching anatomy or for living the immersive experience within a virtual operating room. A total of 3 studies explored the possibility of using MK as a tool for skills learning in bronchoscopy and colonoscopy by means of simulation [110-112].

Various authors explored the possibility of hand tracking [109,126] or instrument tracking [108,121-125] using COTS devices to assess performance in MIS training. From these 2 approaches, Lahanas [120] eventually presented a portable low-cost model of a virtual reality simulator for basic motor skills learning in MIS, which was based on the LMC and capable of tracking instruments. The author also presented face and contrast validity studies. The original forceps tracking problems noted by the author were probably because of the fact that they were black. Problems caused by this color were also described in the study by Oropesa. This issue had already been raised by our group [108].

In the field of simulation for robotic surgery learning, the first studies published [113,115-117] found that the interfaces did not allow robots to be manipulated by gestures. However, the most recent publications [118,119] have suggested that the LMC could be a low-cost solution for creating control interfaces for surgical robots for the purposes of performing operations or training by means of simulation.

### Ethnographic Studies

Ethnographic studies [59,65,78,83,114] deserve a separate mention as they transcend proofs-of-concept and user and prototype testing and approach gesture-based touchless interaction from a holistic viewpoint that includes the social practices of surgery, as well as the way in which medical images and manipulation devices are embedded and made meaningful within the collaborative practices of the surgery [10].

### Requirements for the Future

There was found to be a shortage of objective validation studies (face validity: 1 study; concurrent validity: 3 studies; construct validity: 3 studies; discriminant validity: none; and predictive validity: none) of the different applications developed and presented as prototypes or proofs-of-concept for use in the clinical or teaching field. In teaching, the field of hand gesture–based interfaces should prioritize the following research objectives: first, to transcend studies on technical feasibility and individual hand gesture–based interaction with medical images so as to tackle the issue systematically within a framework of collaborative discussion, as happens in real surgical environments; and second, to conduct experimental studies in simulated surgical environments that allow hand gestures to be validated as a useful tool for touchless interaction in real operating rooms. To that end, the language of hand gestures for medical use would have to be standardized, so that the surgeons’ cognitive load can be reduced. In turn, algorithms should be developed to allow differentiation between intentional and unintentional gestures (spotting) in the small spaces of the operating room. Finally, the problem of temporal segmentation ambiguity (how to define the gesture start and end points) and that of spatial-temporal variability (gestures can vary significantly from one individual to another) must be resolved.

From the range of evidence found, it is possible to infer that, with regard to the use of COTS devices, there is a very interesting field of study for the development and objective validation (contrast, concurrent, discriminant, and predictive validities) of portable low-cost virtual reality simulators for motor skills learning in MIS and robotic surgery. Such simulators will enable surgeons to do presurgical warm-ups anywhere at any time based on 3D reconstructions of specific patients’ images [52,66,70,108]. Thus, surgeons will be able to practice the surgery the night before they are due to perform it from the comfort of their own homes.

Despite the fact that MK was taken off the market in 2017 and that the LMC software only allows tool tracking up to V2 Tracking, the use of interaction with gesture-based virtual environments in the field of simulation identified in this review will enable new COTS devices (ie, the Myo armband) to be explored for skills learning in MIS and robotic surgery.

### Limitations

A number of potential methodological limitations in our systematic review should be discussed. First, our inclusion criteria were limited to English-language publications. Second, although we used the most commonly used search engines in the health field (PubMed, EMBASE, ScienceDirect, Espacenet, OpenGrey, and IEEE) and complemented that by using the snowballing technique to identify relevant articles in the results generated by our search, we may have missed a few articles related to our research question. Finally, there may have been some potential for subjectivity in analyzing the findings, although 2 authors carefully reviewed each study independently and then discussed the results while double-checking each process and subsequently resolved any discrepancies through discussions with the third author whenever necessary.

### Conclusions

As most of the articles identified in this systematic review are proof-of-concept or prototype user testing and feasibility testing studies, we can conclude that the field is still in the exploratory phase in areas requiring touchless manipulation within environments and settings that must adhere to asepsis and antisepsis protocols, such as angiography suites and operating rooms.

Without doubt, COTS devices applied to hand and instrument gesture–based interfaces in the field of simulation for skills learning and training in MIS could open up a promising field to achieve ubiquitous training and presurgical warm-up.

The withdrawal of MK from the market and suspension of the instrument tracking function in the latest LMC software versions constitute threats to the new developments identified in this review. Nevertheless, gesture-based interaction devices are clearly useful for manipulating images in interventional radiology environments or the operating room and for the development of virtual reality simulators for skills training in MIS and robotic surgery.

## Appendix

Multimedia Appendix 1 Summary of included studies evaluating Microsoft Kinect.

Multimedia Appendix 2 Summary of included studies evaluating the Leap Motion Controller.

Multimedia Appendix 3 Summary of included studies evaluating other devices.

## Abbreviations

3D: 3-dimensional
COTS: commercial off-the-shelf
EMBASE: Excerpta Medica dataBASE
IEEE: Institute of Electrical and Electronics Engineers
LMC: Leap Motion Controller
MeSH: Medical Subject Headings
MIS: minimally invasive surgery
MK: Microsoft Kinect
