# Supplementary material for: Use of Commercial Off-The-Shelf Devices for the Detection of Manual Gestures in Surgery: Systematic Literature Review
Source: J Med Internet Res. 2019 May 3;21(5):e11925. doi: 10.2196/11925 (PMC6533048; doi:10.2196/11925)
Supplement: Multimedia Appendix 1 [file jmir_v21i5e11925_app1.pdf]

Table 1. Summary of included studies evaluating MK.

Study	Year	Aim	Type of study	Intervention	Metrics	Sample	Results/Conclusions
[17]	2011	To describe a system for the interactive exploration of medical images through a gesture-controlled interface using MK.	Proof-of-concept.	Manipulation of CT, MRI and Positron emission tomography images.	Not described.	Not described.	Since the interface does not require direct contact or calibration, it is suitable for use in the operating room.
[114]	2011	To explore the potential simplifications derived from using 3D sensors in medical augmented reality applications by designing a low-cost system.	Proof-of-concept.	Augmented reality in Medicine.	Registration time. Registration error.	Not described.	The concept is feasible but the whole process is still too time-consuming to be executed in real time.
[116]	2012	To present an augmented reality magic mirror for anatomy teaching.	Proof-of-concept.	Augmented reality in Medicine. Anatomy education.	Not described.	A hospital and a school.	The system can be used for educational purposes, to improve communication between doctor and patients. A possible use for anatomy teaching in surgery is not mentioned.
[5]	2012	To evaluate the response time and usability (gestures and voice commands) compared to mouse and keyboard controls.	Prototype user testing and feasibility testing.	Manipulation of CT images.	Usability and response time.	2 radiologists and 8 forensic pathologists who recreated 12 images.	Users took 1.4 times longer to recreate an image with gesture control and rated the system 3.4 out of 5 for ease of use in comparison to the keyboard and mouse. The voice recognition system did not work properly.

[102]	2012	To develop a system to allow the surgeon to interact with the standard PACS system during sterile surgical management of orthopedic patients.	Proof-of-concept.	Manipulation of radiological images in orthopedics.	Not described.	Not described.	This is the first example of this technology being used to control digital X-rays in clinical practice.
[101]	2012	To present a sterile method for the surgeon to manipulate images using touchless freehand gestures.	Experiment.	Manipulation of MRI images.	Detection of intent based on contextual cues.<br>Performance in gesture recognition.<br>Navigation and manipulation tasks using MRI images.	9 veterinary surgeons.<br>22 students.	The hypothesis that contextual information integrated with hand trajectory gesture information can significantly improve the overall recognition system performance was validated.<br>The recognition accuracy was 98.7%
[99]	2012	To evaluate an MK-based interaction system for manipulating imaging data using 'Magic Lens visualization'.	Proof-of-concept in the operating room.	Manipulation of radiological images.	Not described.	A laryngoplasty.	The surgeon can manipulate the preoperative information with the intraoperative video and the simulations to correctly place the implant.
[60]	2012	To compare the accuracy and speed of interaction of MK with that of a mouse.<br>To study the performance of the interaction methods in rotation tasks and localization of internal structures in a 3D dataset.	User testing.	Manipulation of radiological images.	Time and accuracy for performing rotation tasks and localization of internal structures.	15 users.	The gesture-based interface outperformed the traditional mouse with respect to time and accuracy in the orientation and rotation task.<br>The mouse was superior in terms of accuracy of localization of internal structures.<br>However, the gesture-based interface was found to have the fastest target

							localization time.
[97]	2012	To develop a user-friendly touchless system for controlling the presentation of medical images based on hand gesture recognition in the operating room.	Proof-of-concept in the operating room.	Manipulation of radiological images in orthopedic surgery.	Not described.	Not described.	The system does not require calibration and was adapted to the surgical environment following the principles of asepsis/antisepsis.
[30]	2012	To present a touchless gesture interface that allows the surgeon to control medical images using hand gestures.	Proof-of-concept and prototype feasibility testing.	Manipulation of CT images.	Not described.	Enucleation of 4 tumors in 3 urology patients.	First description in the literature of a gesture user interface using MK in the operating room in in-vivo surgery, showing that it is an efficient and low-cost solution.
[115]	2012	To develop a low-cost augmented reality interface projected onto a mannequin simulator.	Proof-of-concept.	Augmented reality for education in Medicine.	Not described.	A physical simulator, video projector, Wii Remote and MK.	The manipulations obtained using MK were similar to those described with the Wii.
[92]	2012	To develop a version of a gesture-based system for controlling images.	Proof-of-concept.	Manipulation of MRI images.	Comparison of the rate of recognition of the actions in a clinical case and in a non-clinical case.	Resection of a glioma.	Except for the scanning movement, each movement was recognised with great accuracy.<br>The algorithm can be installed in the clinical area.
[143]	2013	To use MK to operate an automated operating-room light system.	Prototype user testing.	Manipulation of operating room lights.	Ease of learning of the gestures.<br>Time to perform the gestures.<br>Position of the laser points.	18 volunteers.	The gestures were easy to learn and the movement of the light beam was sufficiently precise.

[117]	2013	To create a touchless head tracking system for an immersive virtual operating room.	Proof-of-concept.	Virtual reality for simulation and education in surgery.	The system was tested with respect to the accuracy of the 3D position using a magnetic field based on a position detection device.	A 3D virtual operating room with a virtual operating table.	Using MK, it was possible to implement a very accurate interactive tracking system regardless of the complexity of the virtual reality system.
[103]	2013	To present a new prototype that allows the user to control the OsiriX system with finger gestures using a low-cost depth camera.	Proof-of-concept and prototype feasibility testing.	Manipulation of CT images.	Time required to learn the system.<br>Likert-type scale to rate the intuitiveness of the gestures.	4 forensic pathologists, 1 radiologist and 1 engineer.	On average, 4.5 minutes were required to learn to use the system.<br>Participants rated the intuitiveness of the gestures with 3.8 out of 5 and control of the images with 3.8 out of 5.<br>The low cost of the system makes it affordable for any potential user.
[119]	2013	To present a new immersive surgical training system.	Proof-of-concept and prototype fidelity testing.	Virtual reality for education in surgery.	Not described.	Cholecystectomy training on animal tissue blocks.	Initial feedback from the residents showed that the system is much more effective than the conventional videotaped system.
[87]	2013	To test a speech and gesture-controlled interventional radiology system.	User testing.	Manipulation of CT and angiography images.	The participants rated the convenience of the application and its possible use in everyday clinical routine.	10 radiology residents used commands under different lighting conditions during 18 angiographies and 10 CT-guided punctures.	93% of commands were recognized successfully.<br>Speech commands were less prone to errors than gesture commands.<br>60% of participants would use the application in their routine clinical practice.
[104]	2013	To develop an	Proof-of-	Manipulation of	Measurements	Not described.	The system can be

		image operation system for image manipulation using a motion sensor.	concept.	angiographic images.	of depth, recognition time of the palms, distance for recognition of the hands.		implemented as a useful tool in angiography for controlling image viewing using gestures in the operating room.
[19]	2013	The working hypothesis is that contextual information such as the focus of attention, integrated with gestural information, can significantly improve overall system recognition performance compared with interfaces relying on gesture recognition alone.	Ethnographic study. Experiment. Survey.	Manipulation of MRI images.	Gesture recognition accuracy. Performance of gesture recognition during the tasks.	10 veterinary surgeons. 20 volunteers.	The surgeon's intention to perform a gesture can be accurately recognized by observing environmental cues (context). The hypothesis was validated by a drop in the false positive rate of gesture recognition from 20.76% to 2.33%. A significant rate of reduction of the mean task completion time indicated that the user operates the interface more efficiently with experience. The tracking algorithm occasionally failed in the presence of several people in the camera's field of view.
[61]	2013	To examine the functionality and usability of MK to complete the visualization of 3D anatomical images.	User testing. Survey.	Manipulation of anatomical images.	Accuracy. Time to complete the tasks.	32 participants: Medical students, professors and anatomy laboratory staff.	MK users reached accuracy levels almost identical to those who used a mouse, and spent less time on performing the same tasks. MK showed potential as a device for interaction with medical images.
[118]	2013	To examine usability for navigating	User testing. Survey.	Manipulation of anatomical images.	Time to complete the task.	17 veterinary students.	Improvements should be made to MK before it can be implemented

		through 3D medical images using MK compared to a traditional mouse.		Education.	Accuracy.		as a device for medical use.<br>The preferred method was the mouse.<br>MK has the potential to reduce time on the task.
[13]	2013	To develop a prototype and to examine the feasibility of this new device to help bridge the sterility barrier and eliminate the time and space gap that exists between image review and visual correlation with real-time operative field anatomy.	Proof-of-concept and prototype feasibility testing.	Manipulation of CT and MRI images.	Usability test.<br>Feedback via interviews.<br>Observation of performance during the tasks.	2 MIS procedures and 4 open procedures performed by a surgeon.	The system worked well in a wide range of lighting conditions and procedures.<br>There was an increase in the use of intraoperative image consultation.<br>The gesture library was intuitive and easy to learn.<br>Gestures were mastered within 10 minutes.
[88]	2013	To investigate a solution for manipulating medical images using MK.	Proof-of-concept and prototype feasibility testing.	Manipulation of CT images.	Degree of difficulty of each task.<br>Overall impression of the system.	29 radiologists (diagnostic and interventional).	The potential of the device to enhance image-guided treatment in an interventional radiology suite while maintaining a sterile surgical field was demonstrated.<br>69% of those surveyed believed that the device could be useful in the interventional radiology field.
[125]	2014	To investigate the need for posture and position training during bronchoscopy using a tool	Pilot study.	Analysis of the operator's movements during a bronchoscopy.<br>Education.	Not described.	Not described.	The results highlight the importance of posture during bronchoscopy and the need to implement a training module for the simulator.

		called ETrack					
[73]	2014	To evaluate a new touchless, portable, low-cost 3D measurement system for objective breast assessment.	Concurrent validation study.	Calculation of breast implant volumes.	Volume measurements.	9 silicone implants of known volumes.	The implant volumes were calculated with an error margin of 10%. Reproducibility was satisfactory. The system was validated for clinical use.
[121]	2014	To describe a gesture-controlled 3D teaching tool in which temporal bone anatomy is manipulated without using a mouse or keyboard.<br>To provide a teaching tool for patient-specific anatomy.	Proof-of-concept.	Manipulation of anatomical images.<br>Education.	Not described.	0.15 mm slice thickness cadaveric temporal bone images.	The interactive 3D model developed seems promising as an educational tool.
[89]	2014	To develop hand recognition software based on MK, linked to an interventional CT, to manipulate images.	Feasibility testing	Manipulation of CT images in surgery.	Efficiency, user satisfaction.	10 interventional radiology procedures.<br>1 operator.	Tested on 10 procedures, feasibility was 100%. The system also allowed information to be obtained without using the CT system interface or a third party, and without the loss of operator sterility.
[76]	2014	To present a novel method for training intentional and non-intentional gesture recognition.	Experiment.	Performance of a simulated brain biopsy on a mannequin assisted by images manipulated using gestures.	Accuracy of isolated gesture recognition.<br>Accuracy of continuous gesture recognition.<br>Intention recognition.<br>Time to	19 subjects.	Continuous gesture recognition was successful 92.26% of the time with a reliability of 89.97%. Significant improvements in task completion time were obtained through the context integration effect.

					complete the task.		
[52]	2014	To evaluate two contactless hand tracking systems, the LMC and MK, for their potential to control surgical robots.	Experiment.	Manipulation of robots in surgery.	Comparison of the two systems' range, static positioning error, trajectory accuracy of single finger and hand motions, and latency.	4 trained surgeons.	Neither system has the high level of accuracy and robustness that would be required for controlling medical robots.
[122]	2014	To use a projector for visualization and to provide intuitive means for direct interaction with the information projected onto the surgical surface, using MK to capture the interaction zone and the surgeon's actions on a deformable surface.	Proof-of-concept.	Augmented reality in surgery.	Temporal efficiency of the different parts of the algorithm.	Not described.	The system eliminates the need for the surgeon to look at a location other than the surgical field. It therefore removes distractions and enhances his or her performance.<br>It not only provides the surgeon with medical data during the intervention, but also allows interaction with such information by using gestures.
[10]	2014	To present an ethnographic study of a system based on MK developed to allow touchless control of medical images during vascular surgery.<br>The study aims to go beyond demonstrating	Ethnographic study.	Manipulation of radiological images.	Not described.	Endovascular suite of a large hospital.	With touchless interaction, the visual resources were embedded and made meaningful in the collaborative practices of surgery.<br>The importance of direct and dynamic control of the images by the clinicians in the context of talks and in the context of other

		technical feasibility in order to understand the collaborative practices that emerge from its use in this context.					artefact use is discussed.
[133]	2014	To evaluate a system for manipulating an operating table using gestures.	Prototype user testing.	Manipulation of an operating table.	Effectiveness. Efficiency. User satisfaction.	15 participants.	Major problems were encountered during gesture recognition and with obstruction by other people in the interaction area due to the size and layout of the operating room. The system cannot yet be integrated into a surgical environment.
[67]	2014	To study the technical skills of colonoscopists using MK for motion analysis to develop a tool to guide colonoscopy education and to select discriminative motion patterns.	Construct validity study.	Analysis of the movements of the operator during a colonoscopy.	Seven metrics were analysed to find discriminatory patterns between novice and experienced endoscopists.	10 experienced and 11 novice endoscopists.	Certain types of metric can be used to discriminate between experienced and novice operators.
[79]	2014	To develop a 3D surface imaging system and to assess the accuracy and repeatability on a female mannequin.	Inter-rater reliability study.	Measurement of the surface distances of the breast on a mannequin.	Comparison of surface distances of the breast calculated with the MK system and with a measuring tape. Inter-rater reliability.	A female mannequin.	MK seems to be a useful and feasible system for capturing 3D images of the breast. There was agreement between the measurements obtained by the system and those taken manually with a measuring tape.

[120]	2014	To present a new surgical training system.	Proof-of-concept.	Real-time immersive 3D surgical training. Education.	Rendering speed. Transmission rate. Network transmission rate.	Not described.	Preliminary experiments show that this immersive training system is portable, effective and reliable.
[93]	2014	To present the development and clinical testing of a device that enables intraoperative control of images with hand gestures during neurosurgical procedures.	Proof-of-concept. Initial clinical testing.	Manipulation of MRI images.	Feedback on the performance of the device. Questionnaire for the evaluation of system functionality.	30 neurosurgical operations.	OPECT demonstrated high effectiveness, simplicity of use and precise recognition of the individual user profile. In all cases, surgeons were satisfied with the performance of the device.
(68]	2015	To test whether an automatic motion analysis system could be used to explore if there is a correlation in scope movements and the level of experience of the surgeon performing the bronchoscopy.	Construct validity study. Prospective, comparative study.	Analysis of the operator's movements during a bronchoscopy. Education.	Deviation from the vertical line. Height above the horizontal line. Distance between hands.	11 novice, 9 intermediate and 9 experienced bronchoscopy operators performed 3 procedures each on a bronchoscopy simulator.	The motion analysis system could discriminate between different levels of experience. Automatic feedback on correct movements during self-directed training on simulators might help new bronchoscopists learn how to handle the bronchoscope like an expert.
[53]	2015	To compare two commercial motion sensors (MK and the LMC) to manipulate CT images, in terms of their utility, usability, speed, accuracy and user acceptance.	Two-strand sequential observational study. Qualitative and quantitative descriptive field study using a semi-structured questionnaire.	Manipulation of CT images.	Utility. Usability. Speed. Accuracy. User acceptance.	42 participants: radiologists, surgeons and interventional radiologists.	Marginal to average acceptability of the two devices. MK was found to be more useful and easier to use, but the LMC was more accurate. Further research is required to establish the design specifications, installation guidelines

							and user training requirements to ensure successful implementation in clinical areas.
[86]	2015	To develop an integrated and comprehensive operating room information system compatible with HL7 and DICOM (MediNav). A natural user interface is designed specifically for operating rooms based on MK.	Prototype user testing.	Users tested the application's various modules.	Contextual interviews. Usability satisfaction questionnaire.	A prototype system is tested in a live operating room at an Iranian teaching hospital. 30 general surgeries.	The results of usability tests are promising, and indicate that integration of these systems into a complete solution is the key. Touchless natural user interfaces can help to collect and visualize medical information in a comprehensive manner.
[98]	2015	To propose a novel system to visualize a surgical scene in augmented reality using the different sources of information provided by a C-arm and MK.	Prototype user testing.	Augmented reality in orthopedic surgery.	4 video and depth frames, 20 X-ray shots. Two-fold cross-validation. Questionnaire with a Likert scale.	Simulations of 12 orthopedic procedures. 5 participating clinicians, 3 experienced surgeons, 2 fourth-year medical students.	The system showed promising results with respect to better surgical scene understanding and improved depth perception using augmented reality in simulated orthopedic surgery.
[59]	2016	To explore 3D perception technologies in the operating room.	Ethnographic. Prototype testing.	Detection of the interaction between operating staff and the robot.	Registration, detection accuracy, and registration and accuracy of intention detection.	Not described.	The paper described a supervision system for the operating room that enables intention tracking. The system had low latency, good registration accuracy and high tracking reliability, which make it useful for workflow monitoring, tracking and avoiding collisions between medical robots and operating

							room staff.
[130]	2016	To use MK and color markers to track the position of MIS instruments in real time.	Comparative study between MK and the SinaSim trainer.	Movement of the instrument to position its tip in 81 holes of a Plexiglas plate on 5 occasions.	Data rate (Hz), static noise, static error.	1 user.	Although the new method had inferior accuracy compared to mechanical sensors, its low cost and portability make it a candidate for replacing traditional tracking methods.
[62]	2016	To compare three different interaction modes for image manipulation in a surgery setting: 1) A gesture-controlled approach using MK; 2) verbal instructions to a third party; and 3) direct manipulation using a mouse.	Crossover randomized controlled trial with blocked randomization.	Interaction modes were direct manipulation using a mouse, verbal instructions given to a third party, and gesture-controlled manipulation using MK.	Length of time to complete each task. Trajectory log files were used to calculate performance.	30 physicians and senior medical students	Under the premise that a mouse cannot be used directly during surgery, gesture-controlled approaches were shown to be superior to verbal instructions for image manipulation.
[72]	2017	To evaluate the feasibility, validity, and reliability of the training system for motion parameter and ergonomic analyses between different experience levels of surgeons using the NDI Polaris System and MK camera.	Construct validity, concurrent validity and test-retest reliability. Prospective blinded study.	Tying of intra-corporeal MIS knots.	Time, path length, maximum speed, average speed, number of movements, number of movements per second, angular path.	10 MIS novices, 10 intermediate level and 10 experts.	Validity and reliability of the self-developed sensor and expert model-based MIS training system 'iSurgeon' were established.
[96]	2017	To analyze preoperative breast volume in	Exploratory study.	MK was used to acquire 3D images of the	Patient demographics such as age,	10 patients.	This study showed the feasibility of using fast, simple and inexpensive

		patients with breast cancer in order to predict implant size for reconstruction.		patients' breasts before surgery and after surgery.	body mass index (BMI) and bra size. Used implant sizes. Mastectomy specimen weight.		3D imaging technology for predicting implant size before surgery, although there were significant technical challenges in determining breast volume by surface imaging.
[55]	2017	To evaluate the feasibility of using three different gesture control sensors (MK, the LMC and the Myo armband) to interact in a sterile manner with preoperative data as well as in settings of an integrated operating room during MIS.	Pilot user study.	Two hepatectomies and two partial nephrectomies on an experimental porcine model.	A Likert scale to rate comfort, user friendliness, physical effort, intuitiveness, accuracy, initialization, speed and disconnection.	3 surgeons.	Natural user interfaces are feasible for directly interacting, in a more intuitive and sterile manner, with preoperative images and integrated operating room functionalities during MIS. The combination of the Myo armband and voice commands provided the most intuitive and accurate natural user interface.

CT: Computed Tomography

MRI: Magnetic Resonance Imaging

5. Ebert LC, Hatch G, Ampanozi G, Thali MJ, Ross S. You Can't Touch This: Touch-free Navigation Through Radiological Images. Surg Innov 2012 Nov 6;19(3):301–7. PMID: 22064490
10. O'Hara K, Gonzalez G, Sellen A, Penney G, Varnavas A, Mentis H, et al. Touchless interaction in surgery. Commun ACM 2014 Jan 1;57(1):70–7. doi: 10.1145/2541883.2541899
13. Strickland M, Tremaine J, Brigley G, Law C. Using a depth-sensing infrared camera system to access and manipulate medical imaging from within the sterile operating field. Can J Surg 2013 Jun 1;56(3):E1–6. PMID: 23706851
17. Gallo L, Placitelli AP, Ciampi M. Controller-free exploration of medical image data: Experiencing the Kinect. In: 2011 24th International Symposium on Computer-Based Medical Systems (CBMS) IEEE 2011 p. 1–6. doi: 10.1109/CBMS.2011.5999138
19. Jacob MG, Wachs JP, Packer RA. Hand-gesture-based sterile interface for the operating room using contextual cues for the navigation of radiological images. J Am Med Inform Assoc 2013 Jun;20(e1):e183-6. PMID: 23250787
30. Ruppert GCS, Reis LO, Amorim PHJ, de Moraes TF, da Silva JVL. Touchless gesture user interface for interactive image visualization in urological surgery. World J Urol 2012;30(5):687–91. PMID: 22580994
52. Kim Y, Kim PCW, Selle R, Shademan A, Krieger A. Experimental evaluation of contact-less hand tracking systems for tele-operation of surgical tasks. In: 2014 IEEE International Conference on Robotics and Automation (ICRA) 2014; p. 3502–9. doi: 10.1109/ICRA.2014.6907364
53. Hughes, Nestorov N, Healy N, Sheehy N, O'Hare N. Comparing the utility and usability of the Microsoft Kinect and Leap Motion sensor devices in the context of their application for gesture control of biomedical images. In: European Congress of Radiology. Vienna; 2015. doi: 10.1594/ecr2015/B-1192
55. Sánchez-Margallo FM, Sánchez-Margallo JA, Moyano-Cuevas JL, Pérez EM, Maestre J. Use of natural user interfaces for image navigation during laparoscopic surgery: initial experience. Minim Invasive

Ther Allied Technol 2017 Oct 3;26(5):253–61. PMID: 28349758
59. Beyl T, Schreiter L, Nicolai P, Raczkowsky J, Wörn H. 3D Perception Technologies for Surgical Operating Theatres. Stud Health Technol Inform 2016;220:45–50. PMID: 27046552
60. Kirmizibayrak C, Radeva N, Wakid M, Philbeck J, Sibert J, Hahn J. Evaluation of gesture based interfaces formedical volume visualization tasks. Int J Virtual Real. 2012;11(2):1–13. Available from: http://ro.uow.edu.au/sspapers/1105/
61. Juhnke B, Berron M, Philip A, Williams J, Holub J, Winer E. Comparing the microsoft kinect to a traditional mouse for adjusting the viewed tissue densities of three-dimensional anatomical structures. In: SPIE Proceedings 8673 Medical Imaging 2013: Image Perception, Observer Performance, and Technology Assessment,. 2013. p. 86731M–86731M–10. doi: https://doi.org/10.1117/12.2006994
62. Wipfli R, Dubois-Ferrière V, Budry S, Hoffmeyer P, Lovis C. Gesture-Controlled Image Management for Operating Room: A Randomized Crossover Study to Compare Interaction Using Gestures, Mouse, and Third Person Relaying. PLoS One 2016;11(4):e0153596. PMID: 27082758
67. Svendsen MB, Preisler L, Hillingsoe JG, Svendsen LB, Konge L. Using motion capture to assess colonoscopy experience level. World J Gastrointest Endosc 2014 May 16;6(5):193–9. PMID: 24891932
68. Colella S, Søndergaard Svendsen MB, Konge L, Svendsen LB, Sivapalan P, Clementsen P. Assessment of competence in simulated flexible bronchoscopy using motion analysis. Respiration. 2015;89(2):155–61. PMID: 25591730
72. Kowalewski K-F, Hendrie JD, Schmidt MW, Garrow CR, Bruckner T, Proctor T, et al. Development and validation of a sensor- and expert model-based training system for laparoscopic surgery: the iSurgeon. Surg Endosc 2017 May;31(5):2155–65. PMID: 27604368
73. Henseler H, Kuznetsova A, Vogt P, Rosenhahn B. Validation of the Kinect device as a new portable imaging system for three-dimensional breast assessment. J Plast Reconstr Aesthet Surg 2014 Apr;67(4):483–8. PMID: 24513562
76. Jacob MG, Wachs JP. Context-based hand gesture recognition for the operating room. Pattern Recognit Lett 2014 Jan;36:196–203. doi:https://doi.org/10.1016/j.patrec.2013.05.024
79. Wheat JS, Choppin S, Goyal A. Development and assessment of a Microsoft Kinect based system for imaging the breast in three dimensions. Med Eng Phys 2014 Jun;36(6):732–8. PMID: 24507690
86. Nouei MT, Kamyad AV, Soroush AR, Ghazalbash S. A comprehensive operating room information system using the Kinect sensors and RFID. J Clin Monit Comput 2015 Apr 14;29(2):251–61. PMID: 25017016
87. Hötker AM, Pitton MB, Mildenberger P, Düber C. Speech and motion control for interventional radiology: requirements and feasibility. Int J Comput Assist Radiol Surg 2013 Nov;8(6):997–1002. PMID: 23580026
88. Tan JH, Chao C, Zawaideh M, Roberts AC, Kinney TB. Informatics in Radiology: developing a touchless user interface for intraoperative image control during interventional radiology procedures. Radiographics 2013 Jan;33(2):E61-70. PMID: 23264282
89. Iannessi A, Marcy P-Y, Clatz O, Fillard P, Ayache N. Touchless intra-operative display for interventional radiologist. Diagn Interv Imaging 2014 Mar 1;95(3):333–7. PMID: 24176864
92. Yoshimitsu K, Muragaki Y, Maruyama T, Saito T, Suzuki T, Ikuta S. Clinical trials of the non-touch intraoperative image controllable interface system using KINECT(TM). IJCARS. 2012;7(Suppl 1):S209-10.
93. Yoshimitsu K, Muragaki Y, Maruyama T, Yamato M, Iseki H. Development and initial clinical testing of "OPECT": an innovative device for fully intangible control of the intraoperative image-displaying monitor by the surgeon. Neurosurgery 2014 Mar;10 Suppl 1:46–50; discussion 50. PMID: 24141478
96. Pöhlmann STL, Harkness E, Taylor CJ, Gandhi A, Astley SM. Preoperative implant selection for unilateral breast reconstruction using 3D imaging with the Microsoft Kinect sensor. J Plast Reconstr Aesthetic Surg 2017 Aug 1;70(8):1059–67. PMID: 28595842
97. Klumb F, Dubois-Ferriere V, Roduit N, Barea C, Strgar T, Ahmed K, et al. Virtual manipulation of medical images with hand gestures in the operating room. IJCARS. 2012;7(Suppl 1):S206-207. doi: https://doi.org/10.1007/s11548-017-1588-3
98. Pauly O, Diotte B, Fallavollita P, Weidert S, Euler E, Navab N. Machine learning-based augmented reality for improved surgical scene understanding. Comput Med Imaging Graph 2015 Apr;41:55–60. PMID: 24998759
99. Kirmizibayrak C, Yim Y, Wakid M, Hahn J. Interactive visualization and analysis of multimodal datasets for surgical applications. J Digit Imaging 2012 Dec;25(6):792–801. PMID: 2234991
101. Jacob M, Cange C, Packer R, Wachs J. Intention, context and gesture recognition for sterile MRI navigation in the operating room. In: Alvarez L, Mejail M, Gomez L, Jacobo J, editors. Progress in Pattern Recognition, Image Analysis, Computer Vision, and Applications, volume 7441 of Lecture Notes in Computer Science. Springer Berlin Heidelberg; 2012. p. 220–7. doi: https://doi.org/10.1007/978-3-642-33275-3_27
102. Frame M. A novel system for hands free manipulation of digital X-rays in a sterile environment using consumer electronics and software. IJCARS. 2012;7(Supplement 1):S208. Available from: http://www.caos-international.org/caos-2012/abstracts/024.pdf
103. Ebert LC, Hatch G, Thali MJ, Ross S. Invisible touch—Control of a DICOM viewer with finger gestures

using the Kinect depth camera. J Forensic Radiol Imaging 2013 Jan;1(1):10–4. doi https://doi.org/10.1016/j.jofri.2012.11.006
104. Ishida Y, Ogura T, Hayashi N, Sato M, Okajima M, Doi K. Development of a New Image Operation System with Hand Movements Using a Kinect Sensor for Angiography. In: Radiological Society of North America 2013 Scientific Assembly and Annual Meeting. Chicago, IL; 2013. Available from: https://www.researchgate.net/publication/266096586_Development_of_a_New_Image_Operation_System_with_Hand_Movements_Using_a_Kinect_Sensor_for_Angiography
114. Placitelli AP, Gallo L. 3D point cloud sensors for low-cost medical in-situ visualization. In: International Conference on Bioinformatics and Biomedicine Workshops (BIBMW), 2011 IEEE. Atlanta, GA, USA: IEEE; 2011. p. 596–7. doi: 10.1109/BIBMW.2011.6112435
115. Samosky JT, Wang B, Nelson DA, Bregman R, Hosmer A, Weaver RA. BodyWindows: enhancing a mannequin with projective augmented reality for exploring anatomy, physiology and medical procedures. Stud Health Technol Inform 2012;173:433–9. PMID: 22357032
116. Blum T, Kleeberger V, Bichlmeier C, Navab N. Mirracle: An augmented reality magic mirror system for anatomy education. In: IEEE Virtual Reality 2012. Orange County, CA, USA: IEEE Computer Society; 2012. p. 115–6. PMID: 22357032
117. Dargar S, Nunno A, Sankaranarayanan G, De S. Microsoft kinect based head tracking for life size collaborative surgical simulation environments (LS-CollaSSLE). Stud Health Technol Inform 2013;184:109–13. PMID: 23400140
118. Juhnke B. Evaluating the Microsoft Kinect compared to the mouse as an effective interaction device for medical imaging manipulations. Iowa State University, Graduate Theses and Dissertations; 2013. Available from: https://lib.dr.iastate.edu/cgi/viewcontent.cgi?article=4362&context=etd
119. Guo X, Lopez LD, Yu Z, Steiner K V, Barner KE, Bauer TL, et al. A portable immersive surgery training system using RGB-D sensors. Stud Health Technol Inform 2013 Jan;184:161–7. PMID: 23400150
120. Yang Y, Guo X, Yu Z, Steiner K V, Barner KE, Bauer TL, et al. An immersive surgery training system with live streaming capability. Stud Health Technol Inform 2014 Jan;196:479–85. PMID: 24732560
121. Hochman JB, Unger B, Kraut J, Pisa J, Hombach-Klonisch S. Gesture-controlled interactive three dimensional anatomy: a novel teaching tool in head and neck surgery. J Otolaryngol Head Neck Surg 2014 Oct 7;43(1):38. PMID 25286966
122. Kocev B, Ritter F, Linsen L. Projector-based surgeon–computer interaction on deformable surfaces. Int J Comput Assist Radiol Surg [Internet]. 2014 Jul 26 [cited 2015 Oct 20];9(2):301–12. PMID: 23888316
125. Coles TR, Cao CG, Dumas C. ETrack: An affordable Ergonomic assessment tool for surgical settings. SAGES abstract archives. 2014. Available from: http://www.sages.org/meetings/annual-meeting/abstracts-archive/etrack-an-affordable-ergonomic-assessment-tool-for-surgical-settings/
130. Owlia M, Khabbazan M, Mirbagheri MM, Mirbagheri A. Real-time tracking of laparoscopic instruments using kinect for training in virtual reality. Conf Proc . Annu Int Conf IEEE Eng Med Biol Soc IEEE Eng Med Biol Soc Annu Conf 2016 Aug;2016:3945–8. PMID: 28269148
133. Schröder S, Loftfield N, Langmann B, Frank K, Reithmeier E. Contactless operating table control based on 3D image processing. Conf Proc . Annu Int Conf IEEE Eng Med Biol Soc IEEE Eng Med Biol Soc Annu Conf 2014;2014:388–92. PMID: 25569978
143. Hartmann F, Schlaefer A. Feasibility of touch-less control of operating room lights. Int J Comput Assist Radiol Surg 2013 Mar;8(2):259–68. PMDI:22806717
