# Supplementary material for: Use of Commercial Off-The-Shelf Devices for the Detection of Manual Gestures in Surgery: Systematic Literature Review
Source: J Med Internet Res. 2019 May 3;21(5):e11925. doi: 10.2196/11925 (PMC6533048; doi:10.2196/11925)
Supplement: Multimedia Appendix 2 [file jmir_v21i5e11925_app2.pdf]

Table 2. Summary of included studies evaluating the LMC.

Study	Year	Aim	Type of study	Intervention	Metrics	Sample	Results/Conclusions
[90]	2014	To evaluate the implementation of a low-cost device for touchless PACS control in an interventional radiology suite. To demonstrate that interaction with gestures can decrease the duration of the procedures, the risk of re-intervention, and improve technical performance.	Proof-of-concept and prototype feasibility testing.	Manipulation of images in interventional radiology.	Not described.	Interventional radiology suite.	The LMC is a feasible, portable and low-cost alternative to other touchless PACS interaction systems. A decrease in the need for re-intervention was reported, but no explanation was given of how it was measured.
[83]	2014	To present the first experience of using new systems for image control in the operating room: the LMC and OsiriX.	Proof-of-concept.	Manipulation of CT and MRI images.	Average training time.	2 general surgeons, 1 urologist, 3 orthopedic surgeons and 2 surgeons	The average training time was 5 minutes. The system is very cost-effective, efficient and prevents contamination during surgery. First experience of using the LMC to control CT and MRI images during surgery.
[126]	2014	To validate the possibility of performing precise telesurgical tasks by means of the LMC.	Comparative study of the Sigma.7 electro-mechanical device and the LMC.	Peg transferring task and answering a questionnaire. The success rate of peg transfers.	Time to complete the task. Satisfaction questionnaire.	10 researchers.	The results allowed the authors to confirm that fine tracking of the hand could be performed with the LMC. The observed performance of the optical interface proved to be comparable to that of

							traditional electro-mechanical devices.
[105]	2014	To describe a piece of software for image processing with OsiriX using finger gestures.	Proof-of-concept.	Manipulation of radiological images.	Not described.	Not described.	It is possible to implement gesture control of medical devices with low-cost, minimal resources.<br>The device is very sensitive to surface dirt and this affects performance.<br>The device favors the occlusion phenomenon.
[52]	2014	To evaluate two contactless hand tracking systems, the LMC and MK, for their potential to control surgical robots.	Experiment.	Manipulation of robots in surgery.	Comparison of the two systems' range, static positioning error, trajectory accuracy of single finger and hand motions, and latency.	4 trained surgeons.	Neither system has the high level of accuracy and robustness that would be required for controlling medical robots.
[132]	2014	To evaluate the LMC for simple 2D interaction and the action of entering a value.	Proof-of-concept and prototype testing.	Manipulation of medical information and operating room lights.	2D interaction on entering a value.	A 90-minute conference on computer science and untrained users.	The user cases should be carefully classified and the most appropriate gestures for each application should be detected and implemented.<br>Optimal lighting conditions for the LMC have still not been evaluated since unwanted light with deterioration of the IR light emitted may lead to a reduction in the recognition rate.
[63]	2014	To compare the average time required by the	Observational study.	Manipulation of angiographic images.	Comparison of the average time required	11 radiologists who observed a simulated clinical	After a practice time of 30 minutes, the average operation time

		conventional method using a mouse and an operating method with a finger-motion sensor.			with a mouse and the LMC.	case.	by the finger method was significantly shorter than that by the mouse method.
[14]	2014	To develop a workstation that allows intraoperative touchless control of diagnostic and surgical images in dentistry.	Prototype user testing.	Manipulation of radiological images.	Not described.	2 surgeons.<br>A case series of 11 dental surgery procedures.	The system performed very well.<br>Its low cost favors its incorporation into clinical facilities of developing countries, reducing the number of staff required in operating rooms.
[106]	2014	To propose an interface to control hand gestures and gestures with hand-held tools. In this approach, hand-held tools can become gesture devices that the user can use to control the images.	Prototype user testing.	Manipulation of ultrasound images.	Time to complete the task.<br>Questionnaire with Likert-type scale.	12 participants.	Users were able to significantly improve their performance with practice.
[85]	2014	To develop a software application for the manipulation of a 3D pancreatic or liver tumor model by using CT and real-time elastography data.	Proof-of-concept.	Manipulation of CT and real-time elastography images.	Not described.	15 patients with liver cancer and 10 patients with pancreatic cancer.	A 3D model of liver and pancreatic tumors was successfully implemented with a hands-free interaction device suitable for sterile environments and for aiding diagnostic or therapeutic interventions.
[127]	2014	To present a new gesture recognition system for	Proof-of-concept.	Manipulation of robots in surgery.	Not described.	2 surgical robots in a virtual simulator.	The device provided satisfactory accuracy and speed.<br>It requires a more

		manipulating two surgical robots in a virtual simulator.					complete API.
[108]	2014	To propose a web-based interface to retrieve medical images using gestures.	User testing. Pilot study.	Manipulation of radiological images.	Not described.	2 users.	User feedback was positive. Users reported fatigue with prolonged use of gestures. Additional studies are required to validate the interface.
[91]	2015	To describe the use of the LMC for image manipulation during hepatic transarterial chemoembolization and internal radiotherapy procedures.	Proof-of-concept.	Manipulation of images in interventional radiology.	Not described.	Not described.	Gesture-based imaging control may lead to increased efficacy and safety with decreased radiation exposure during hepatic transarterial chemoembolization procedures.
[53]	2015	To compare two commercial motion sensors (MK and the LMC) to manipulate CT images, in terms of their utility, usability, speed, accuracy and user acceptance.	Two-strand sequential observational study. Qualitative and quantitative descriptive field study using a semi-structured questionnaire.	Manipulation of CT images.	Utility. Usability. Speed. Accuracy. User acceptance.	42 participants: radiologists, surgeons and interventional radiologists.	Marginal to average acceptability of the two devices. MK was found to be more useful and easier to use, but the LMC was more accurate. Further research is required to establish the design specifications, installation guidelines and user training requirements to ensure successful implementation in clinical areas.
[109]	2015	To evaluate a new method for image manipulation using a motion	Observational study. User testing and proof-of-concept.	Manipulation of radiological images in dentistry.	Operating time.	14 students. 6 images.	Using the system, several processes can be performed quickly with finger movements.

		sensor.					Using gestures was significantly superior to using a mouse in terms of time.
[110]	2015	To develop a new system for manipulating images using a motion sensor.	Observational study.	Manipulation of radiological images in dentistry.	Time required to view a series of images.	14 students.<br>25 images.	The operation time with the LMC was significantly shorter than with the conventional method using a mouse.
[123]	2016	To design a virtual 3D online environment for motor skills learning in MIS using exercises from the MISR-VR.<br>The environment is designed in Unity, and the LMC is used as the device for interaction with the MIS forceps.	Letter to the editor.	None.	Not described.	Not described	If it can be shown that 3D online environments mediated by natural user interfaces enable motor skills learning in MIS, a new field of research and development in the area of surgical simulation will be opened up.
[78]	2016	Patent for accurate 3D instrument positioning.	Patent.	None.	Not described.	Not described	Representing, on an output display, 3D positions and orientations of an instrument while medical procedures are being performed.
[94]	2016	To describe the configuration for using the LMC in neurosurgery for image manipulation during a surgical procedure.	User testing.	Manipulation of images during a surgical procedure.	Not described.	Resection of a meningioma and sarcoma surgery.	The learning curve only took 30 minutes. Although the main disadvantage was the lack of standardization of the gestures, the LMC is a low-cost, reliable and easily personalized device for controlling images in the surgical environment.

[124]	2016	To develop skills in students and professionals using computer simulation technologies based on hand gesture capture systems.	User testing.	Description of the virtual environment.	Not described.	Not described.	Simulation and new gesture recognition technologies open up new possibilities for the generation of computer-mediated procedures for medical training.
[111]	2016	To present a gesture-controlled projection display that enables a direct and natural physician-machine interaction during CT-based interventions.	User testing (pilot and main).	8 tasks manipulating CT images.	A Likert scale and a usability questionnaire.	12 participants (biomedical engineers, medical students and radiologists).	Gesture recognition is robust, although there is potential for improvement. The gesture training times are less than 10 minutes, but vary considerably between study participants.
[112]	2016	To develop an anatomy learning system using the LMC.	User testing.	Manipulation of 220 anatomical images.	User satisfaction questionnaire based on a Likert scale.	30 students and lecturers from an anatomy department.	The anatomy learning system using the LMC was successfully developed and it is suitable and acceptable as a support tool in an anatomy learning system.
[77]	2016	To study the possibility of tracking laparoscopic instruments using the LMC in a box trainer.	Experiment.	Three static experiments and one dynamic experiment.	Static long precision, static short precision, static distance, and dynamic distance precision.	1 user.	The LMC had acceptable precision for tracking laparoscopic instruments in a box trainer.
[69]	2016	To assess the potential of the LMC to track the movement of hands using MIS instruments.	Construct validity, concurrent validity. Comparative study with the	Passing a thread through pegs using the eoSim simulator.	Time to complete the task, path distance, speed, acceleration, motion	3 experts and 10 novices.	The LMC is able to track the movement of hands using instruments in a MIS box simulator. Construct validity was

			InsTrac.		smoothness, and distance between the instruments.		demonstrated. Concurrent validity was only demonstrated for time and instrument path distance. A number of limitations to the tracking method used by LMC have been identified.
[128]	2016	To explore the use of the LMC in endonasal pituitary surgery and to compare it with the Phantom Omni.	Comparative study between the LMC and the Phantom Omni.	16 resections of simulated pituitary gland tumors using a robot manipulated by the Phantom Omni and by the LMC.	Percentage of tumor resection and procedure duration.	3 neurosurgeons.	Users were able to achieve a very similar percentage of resection and procedure duration using the LMC.
[113]	2016	To try to interact with medical images via a web browser using the LMC.	Prototype user testing.	Rotation, panning, scaling and selection of slices of a reconstructed 3D model based on CT or MRI.	Not described.	1 user.	It is feasible to build this system and interaction can be carried out in real time.
[54]	2017	To analyze the value of two gesture input modalities (the Myo armband and the LMC) versus two clinically established methods (task delegation and joystick control).	User study. Comparative study.	Simulating a diagnostic neuroradiological vascular treatment with two frequently used interaction tasks in an experimental operating room.	Task completion time, perceived task difficulty, and subjective workload.	10 neuroradiologists	Novel input modalities have the potential to carry out single tasks more efficiently than clinically established methods.
[64]	2017	To investigate the potential of a virtual reality simulator for the assessment of	Face and construct validity.	Three basic tasks: camera navigation, instrument navigation, and	Time, path length, and errors.	2 groups of surgeons (28 experts and 21 novices).	This study provides evidence of the potential use of the LMC for assessing basic laparoscopic

		basic laparoscopic skills, based on the LMC		two-handed operation.			skills. The proposed system allows the dexterity of hand movements to be evaluated.
[55]	2017	To evaluate the feasibility of using three different gesture control sensors (MK, the LMC and the Myo armband) to interact in a sterile manner with preoperative data as well as in settings of an integrated operating room during MIS.	Pilot user study.	Two hepatectomies and two partial nephrectomies on an experimental porcine model.	A Likert scale to rate comfort, user friendliness, physical effort, intuitiveness, accuracy, initialization, speed and disconnection.	3 surgeons	Natural user interfaces are feasible for directly interacting, in a more intuitive and sterile manner, with preoperative images and integrated operating room functionalities during MIS.<br>The combination of the Myo armband and voice commands provided the most intuitive and accurate natural user interface.
[70]	2017	To evaluate the LMC as a tool for the objective measurement and assessment of surgical dexterity among users at different experience levels.	Construct validity study.	Surgical knot tying and manual transfer of objects.	Path length, number of movements and total time.	11 participants.	The study showed 100% accuracy in discriminating between expert and novice performances.
[71]	2017	To design an affordable and easily accessible endoscopic third ventriculostomy simulator based on the LMC, and to compare it to the NeuroTouch for its usability and training effectiveness.	Concurrent and construct validity study.	Four ellipsoid practice targeting tasks and 36 ventricle targeting tasks.	Task speed and accuracy.	16 novice users and 2 expert neurosurgeons	An easy-access simulator was created, which has the potential to become a training tool and a surgical training assessment tool.<br>This system can be used for planning procedures using patient datasets.
[129]	2018	To present the	Comparative	Comparison of	Metric-based.	3 operators.	With contactless

		LMC as a novel control device to manipulate the RAVEN-II robot.	study between the LMC and the electro-mechanical Sigma.7.	peg manipulations during a training task with a contact-based device (Sigma.7).	A novel spatiotemporal trajectory clustering.		control, manipulability is not as good as it is with contact-based control. Complete control of the surgical instruments is feasible. This work is promising for the development of future human-machine interfaces dedicated to robotic surgical training systems.
[80]	2018	To evaluate the effect of using virtual reality surgery on the self-confidence and knowledge of surgical residents (the LMC and Oculus Rift).	Multisite, single-blind, parallel, randomized controlled trial.	The study group used the virtual reality surgery application. The control group used similar content in a standard presentation.	Self-assessment scores for trainee confidence using a Likert scale and an objective assessment of cognitive skills.	95 residents from 7 dental schools.	Immersive virtual reality experiences improve the knowledge and self-confidence of the surgical residents.
[65]	2018	To develop and validate a novel training tool for Le Fort I osteotomy based on immersive virtual reality (the LMC and Oculus Rift).	Face and content validity.	A pre-intervention questionnaire to understand training needs and a post-intervention feedback questionnaire.	Realism, usability, efficacy and applicability to orthognathic surgical training.	7 consultant oral and maxillofacial surgeons.	The results confirmed the clinical applicability of virtual reality for delivering training in orthognathic surgery.
[95]	2018	To investigate the feasibility and practicability of a low-cost multimodal head-mounted display system in neuroendoscopic surgery (the LMC and Oculus Rift).	Proof-of-concept in the operating room.	Ventriculocysto-cisternostomy. Ventriculostomy. Tumoral biopsy.	Learning curve. Operation time. Surgeon fatigue. Comfort with the device.	21 patients with ventricular diseases. 1 neurosurgeon.	The head-mounted display system is feasible, practical, helpful, and relatively cost efficient in neuroendoscopic surgery.

CT: Computed Tomography

MRI: Magnetic Resonance Imaging

14. Rosa GM, Elizondo ML. Use of a gesture user interface as a touchless image navigation system in dental

surgery: Case series report. Imaging Sci Dent 2014 Jun;44(2):155–60. PMID: 24944966
52. Kim Y, Kim PCW, Selle R, Shademan A, Krieger A. Experimental evaluation of contact-less hand tracking systems for tele-operation of surgical tasks. In: 2014 IEEE International Conference on Robotics and Automation (ICRA) 2014; p. 3502–9. doi: 10.1109/ICRA.2014.6907364
53. Hughes, Nestorov N, Healy N, Sheehy N, O'Hare N. Comparing the utility and usability of the Microsoft Kinect and Leap Motion sensor devices in the context of their application for gesture control of biomedical images. In: European Congress of Radiology. Vienna; 2015. doi: 10.1594/ecr2015/B-1192
54. Hettig J, Saalfeld · Patrick, Luz M, Becker M, Skalej M, Hansen C. Comparison of gesture and conventional interaction techniques for interventional neuroradiology. Int J CARS 2017;12:1643–53. PMID: 28120179
55. Sánchez-Margallo FM, Sánchez-Margallo JA, Moyano-Cuevas JL, Pérez EM, Maestre J. Use of natural user interfaces for image navigation during laparoscopic surgery: initial experience. Minim Invasive Ther Allied Technol 2017 Oct 3;26(5):253–61. PMID: 28349758
63. Ogura T, Sato M, Ishida Y, Hayashi N, Doi K. Development of a novel method for manipulation of angiographic images by use of a motion sensor in operating rooms. Radiol Phys Technol 2014;7(2):228–34. PMID: 24609904
64. Lahanas V, Loukas C, Georgiou K, Lababidi H, Al-Jaroudi D. Virtual reality-based assessment of basic laparoscopic skills using the Leap Motion controller. Surg Endosc 2017 Dec 2;31(12):5012–23. PMID: 28466361
65. Pulijala Y, Ma M, Pears M, Peebles D, Ayoub A. An innovative virtual reality training tool for orthognathic surgery. Int J Oral Maxillofac Surg 2018 Sep 1;47(9):1199–205. PMID: 29398172
69. Partridge RW, Brown FS, Brennan PM, Hennessey IA, Hughes MA. The LEAP™ gesture interface device and Take-Home laparoscopic simulators: A study of construct and concurrent validity. Surg Innov. 2016;23(1):70–7. PMID: 26178693
70. Sun X, Byrns S, Cheng I, Zheng B, Basu A. Smart Sensor-Based Motion Detection System for Hand Movement Training in Open Surgery. J Med Syst 2017 Feb;41(2):24. PMID: 28000118
71. Wright T, de Ribaupierre S, Eagleson R. Design and evaluation of an augmented reality simulator using leap motion. Healthc Technol Lett 2017 Oct;4(5):210–5. PMID: 29184667
77. Oropesa I, de Jong TL, Sánchez-González P, Dankelman J, Gómez EJ. Feasibility of tracking laparoscopic instruments in a box trainer using a Leap Motion Controller. Measurement 2016;80:115–24. doi: https://doi.org/10.1016/j.measurement.2015.11.018
78. Beck P. Accurate Three-dimensional Instrument Positioning [Internet]. US; 2016. p. 1–26. Available from: http://www.freepatentsonline.com/20160354152.pdf
80. Pulijala Y, Ma M, Pears M, Peebles D, Ayoub A. Effectiveness of Immersive Virtual Reality in Surgical Training—A Randomized Control Trial. J Oral Maxillofac Surg 2018 May 1;76(5):1065–72. PMID: 29104028
83. Bizzotto N, Costanzo A, Bizzotto L, Regis D, Sandri A, Magnan B. Leap Motion Gesture Control With OsiriX in the Operating Room to Control Imaging: First Experiences During Live Surgery. Surg Innov 2014;21(6):655–6. PMID: 24742500
85. Streba CT, Gheonea IA, Streba L, Sandulescu LD, Saftoiu A, Gheone DI. Sa1976 Virtual Palpation Model -Combining Spiral CT and Elastography Data: a Proof-of-Concept Study. Gastroenterology 2014;146(5):S-344-S-345. Available from: http://linkinghub.elsevier.com/retrieve/pii/S0016508514612452
90. Bercu Z, Patil V, Patel R, Kim E, Nowakowski F, Lookstein R, et al. Leap Motion Controller implementation for touchless PACS integration in the interventional radiology suite. Cardiovasc Intervent Radiol. 2014;37(Suppl. 1). doi: https://doi.org/10.1007/s00270-013-0835-4
91. Bercu ZL, Patil V V., Patel RS, Kim E, Nowakowski, S. F., Lookstein RA, Fischman AM. Use of hands free gesture-based imaging control for vessel identification during hepatic transarterial chemoembolization and selective internal radiotherapy procedures. J Vasc Interv Radiol. 2015;26(2 Supplement):S186–7. doi: https://doi.org/10.1016/j.jvir.2014.12.499
94. Di Tommaso L, Aubry S, Godard J, Katranji H, Pauchot J. [A new human machine interface in neurosurgery: The Leap Motion(®). Technical note regarding a new touchless interface]. Neurochirurgie 2016 Jun;62(3):178–81. PMID: 27234915
95. Xu X, Zheng Y, Yao S, Sun G, Xu B, Chen X. A low-cost multimodal head-mounted display system for neuroendoscopic surgery. Brain Behav 2018 Jan;8(1):e00891. PMID: 29568688
105. Ebert LC, Flach PM, Thali MJ, Ross S. Out of touch - A plugin for controlling OsiriX with gestures using the leap controller. J Forensic Radiol Imaging 2014;2(3):126–8. doi: https://doi.org/10.1016/j.jofri.2014.05.006
106. Rossol N, Cheng I, Rui Shen, Basu A. Touchfree medical interfaces. Conf Proc . Annu Int Conf IEEE Eng Med Biol Soc IEEE Eng Med Biol Soc Annu Conf 2014 Jan;2014:6597–600. PMID: 25571508
108. Widmer A, Schaer R, Markonis D, Müller H. Gesture interaction for content--based medical image retrieval. In: ACM International Conference on Multimedia Retrieval. Glasgow; 2014. doi: 10.1145/2578726.2578804
109. Ogura T, Sato M, Kadowaki Y, Yasumoto Y, Okajima M, Tsutsumi S, et al. Development of a new method for manipulation of dental images using a motion sensor in dentistry. In: European Congress of

Radiology [Internet]. Vienna, Austria: European Congress of Radiology 2015. doi: http://dx.doi.org/10.1594/ecr2015/C-0251
110. Sato M, Ogura T, Yasumoto Y, Kadowaki Y, Hayashi N, Doi K. Development of an image operation system with a motion sensor in dental radiology. Radiol Phys Technol 2015 Jul;8(2):243–7. PMID: 25896229
111. Mewes A, Saalfeld P, Riabikin O, Skalej M, Hansen C. A gesture-controlled projection display for CT-guided interventions. Int J Comput Assist Radiol Surg 2016 Jan;11(1):157–64. PMID: 25958060
112. Nainggolan FL, Siregar B, Fahmi F. Anatomy learning system on human skeleton using Leap Motion Controller. In: 2016 3rd International Conference on Computer and Information Sciences (ICCOINS) IEEE; 2016; p. 465–70. doi: 10.1109/ICCOINS.2016.7783260
113. Virag I, Stoicu-Tivadar L, Crisan-Vida M. Gesture-based interaction in medical interfaces. In: 2016 IEEE 11th International Symposium on Applied Computational Intelligence and Informatics (SACI) IEEE; 2016; p. 519–23. doi: 10.1109/SACI.2016.7507339
123. Alvarez-Lopez F, Maina MF, Saigí-Rubió F. Natural User Interfaces: Is It a Solution to Accomplish Ubiquitous Training in Minimally Invasive Surgery? Surg Innov 2016 Aug;23(4):429-30. PMID: 27009688
124. Juanes JA, Gómez JJ, Peguero PD, Ruisoto P. Digital Environment for Movement Control in Surgical Skill Training. J Med Syst 2016 Jun;40(6):133. PMID: 27091754
126. Despinoy F, Sánchez A, Zemiti N, Jannin P, Poignet P. Comparative Assessment of a Novel Optical Human-Machine Interface for Laparoscopic Telesurgery. In Springer, Cham; 2014; p. 21–30. doi: https://doi.org/10.1007/978-3-319-07521-1_3
127. Vargas HF, Vivas OA. Gesture recognition system for surgical robot's manipulation. In: 2014 XIX Symposium on Image, Signal Processing and Artificial Vision IEEE; 2014; p. 1–5. doi: 10.1109/STSIVA.2014.7010172
128. Travaglini TA, Swaney PJ, Weaver KD, Webster III RJ. Initial Experiments with the Leap Motion as a User Interface in Robotic Endonasal Surgery. In: Robotics and mechatronics : proceedings of the 4th IFToMM International Symposium on Robotics and Mechatronics IFToMM International Symposium on Robotics and Mechatronics (4th : 2015 : Poitiers, France) 2016. p. 171–9. PMID: 26752501
129. Despinoy F, Zemiti N, Forestier G, Sánchez A, Jannin P, Poignet P. Evaluation of contactless human–machine interface for robotic surgical training. Int J Comput Assist Radiol Surg 2018 Jan 15;13(1):13–24. PMID: 28914409
132. Mauser S, Burgert O. Touch-free, gesture-based control of medical devices and software based on the leap motion controller. Stud Health Technol Inform 2014;196:265–70. PMID: 24732520
