# Supplementary material for: Use of Commercial Off-The-Shelf Devices for the Detection of Manual Gestures in Surgery: Systematic Literature Review
Source: J Med Internet Res. 2019 May 3;21(5):e11925. doi: 10.2196/11925 (PMC6533048; doi:10.2196/11925)
Supplement: Multimedia Appendix 3 [file jmir_v21i5e11925_app3.pdf]

Table 3. Summary of included studies evaluating other devices.

Study	Year/ Device	Aim	Type of study	Intervention	Metrics	Sample	Results/Conclusions
[82]	2004 Camera with CMOS sensor	To propose an architecture for a real-time multimodal system to provide a touchless user interface in surgery.	Prototype user testing.	Gesture detection in computer-assisted surgery.	Time to perform the tasks. Questionnaire about the experience.	16 subjects in a simulated setting. Video-assisted surgery.	The preliminary results show good usability and rapid learning. The average time to click anywhere on the screen was less than 5 seconds. Lighting conditions affected the performance of the system. The surgeon showed strong interest in the system and satisfactorily assessed the use of gestures within the operating room.
[100]	2006 Webcam	To describe a vision-based system that can interpret gestures in real time to manipulate objects within a medical data visualization environment.	Prototype user testing.	Manipulation of medical data (radiology images and selection of medical records) and movement of objects and windows on the screen.	Performance rate in 4 tasks.	1 user.	The system implemented in a sterile environment demonstrated performance rates between 95% and 100%.
[27]	2008 Canon VC-C4 color camera	To describe a vision-based gesture capture system that interprets gestures in real time to manipulate medical images.	Beta testing during a surgical procedure. Experiment.	A beta test of a system prototype was conducted during a live brain biopsy operation, where neurosurgeons were able to browse through MRI images of the	Gesture recognition accuracy. Task learning. Excess gestures used. Rotation accuracy. Beta test during a	10 inexperienced users. 1 experienced user.	Gesture recognition accuracy was 96%. For every repeat of trials, the task completion time decreased by 28% and the learning curve levelled off at the 10th attempt. The gestures were

				patient's brain using the sterile hand gesture interface.	biopsy.		learned very quickly and there was a significant decrease in the number of excess gestures.<br>Rotation accuracy was reasonable.<br>The surgeons rated the system as easy to use, with a rapid response, and useful in the surgical environment.
[26]	2008<br>Canon VC-C4 camera	To evaluate the Gestix system.	Prototype user testing.	Manipulation of MRI images during a neurosurgical biopsy.	Contextual interview.<br>Individual interview.<br>Subjective satisfaction questionnaire.	1 neurosurgical biopsy.	The system setup time was 20 minutes.<br>The surgeons found the Gestix system easy to use, with a rapid response, and easy to learn.<br>The system does not require the use of wearable devices.
[56]	2011<br>Interaction with gestures in general	Fieldwork focusing on work practices and interactions in an angiography suite and on understanding the collaborative work practices in terms of image production and use.	Ethnographic study of minimally invasive image-guided procedures within an interventional radiology department.	Manipulation of radiological images.	Not described.	Angiography suite of a large hospital in the United Kingdom.	The paper discusses the implications of the findings in the work environment for touchless interaction technologies, and suggests that these will be of importance in considering new input techniques in other medical settings.
[74]	2012<br>Commercial video camera	To describe the development of Gestonurse, a robotic system for surgical instruments.	Proof-of-concept.	Surgical instrumentation using a robot.	Percentage gesture recognition.<br>Speed.	A simulated surgical environment.	95% of gestures were recognized correctly.<br>The system was only 0.83 seconds slower when compared with the performance of a

							human instrument handler.
[57]	2012 Touchless interaction systems in general	To understand and use common practices in the surgical setting from a proxemics point of view to uncover implications for the design of touchless interaction systems.<br>The aim is to think of touchlessness in terms of its spatial properties.<br>What does spatial separation imply for the introduction of the touchless control of medical images?	Ethnographic study.	Field observations of work practices in neurosurgery.	Not described.	A neurosurgery operating room.	Alternative ideas, such as multiple cameras, are the kind of solution that these findings suggest.<br>Such reflections and considerations can be revealed through careful analysis of the spatial organization of activity and proxemics of particular interaction mechanisms.<br>However, it is very important to study current practice in order to speculate about new systems, because they in turn may alter practice.
[75]	2013 Webcam	To present a system for tracking the movement of MIS instruments based on an orthogonal webcam system installed in a physical	Experiment.	Recording the movements of the instrument within an imaginary cube.	Linear triangulation method.	Not described.	The results showed a resolution of 0.616 mm on each axis of work, linearity and repeatability in motion tracking, as well as automatic detection of the 3D position of the tip of the surgical instruments with sufficient accuracy.<br>The system is a low-

		simulator.					cost and portable alternative to traditional instrument tracking devices.
[55]	2017 MK, the LMC, the Myo armband and voice control	To evaluate the feasibility of using three different gesture control sensors (MK, the LMC and the Myo armband) to interact in a sterile manner with preoperative data as well as in settings of an integrated operating room during MIS.	Pilot user study.	Two hepatectomies and two partial nephrectomies on an experimental porcine model.	A Likert scale to rate comfort, user friendliness, physical effort, intuitiveness, accuracy, initialization, speed and disconnection	3 surgeons	Natural user interfaces are feasible for directly interacting, in a more intuitive and sterile manner, with preoperative images and integrated operating room functionalities during MIS. The combination of the Myo armband and voice commands provided the most intuitive and accurate natural user interface.
[54]	2017 The Myo armband and the LMC	To analyze the value of two gesture input modalities (the Myo armband and the LMC) versus two clinically established methods (task delegation and joystick control).	User study. Comparative study.	Simulating a diagnostic neuroradiological vascular treatment with two frequently used interaction tasks in an experimental operating room.	Task completion time, perceived task difficulty, and subjective workload.	10 neuroradiologists	Novel input modalities have the potential to carry out single tasks more efficiently than clinically established methods.

CT: Computed Tomography

MRI: Magnetic Resonance Imaging

26. Wachs J, Stern H, Edan Y, Gillam M, Handler J, Feied C, et al. A gesture-based tool for sterile browsing of radiology images. J Am Med Inform Assoc 2008 Jan;15(3):321–3. PMID: 18451034
27. Wachs J, Stern H, Edan Y, Gillam M, Feied C, Smithd M, et al. Real-time hand gesture interface for browsing medical images. Int J Intell Comput Med Sci Image Process 2008;2(1):15–25. doi: https://doi.org/10.1080/1931308X.2008.10644149
54. Hettig J, Saalfeld · Patrick, Luz M, Becker M, Skalej M, Hansen C. Comparison of gesture and conventional interaction techniques for interventional neuroradiology. Int J CARS 2017;12:1643–53. PMID: 28120179
55. Sánchez-Margallo FM, Sánchez-Margallo JA, Moyano-Cuevas JL, Pérez EM, Maestre J. Use of natural

user interfaces for image navigation during laparoscopic surgery: initial experience. Minim Invasive Ther Allied Technol 2017 Oct 3;26(5):253–61. PMID: 28349758

56. Johnson R, O'Hara K, Sellen A, Cousins C, Criminisi C. Exploring the Potential for Touchless Interaction in Image-Guided Interventional Radiology. In: 29th International Conference on Human Factors in Computing Systems ACM CHI 2011. Vancouver; 2011. p. 3323–32. doi: 10.1145/1978942.1979436

57. Mentis H, O'Hara K, Sellen A, Rikin Trivedi R. Interaction proxemics and image use in neurosurgery. In: Proceedings of the SIGCHI Conference on Human Factors in Computing Systems (CHI '12). New York: ACM Conference on Computer-Human Interaction; 2012. p. 927–36. doi:10.1145/2207676.2208536

74. Jacob M, Li Y-T, Akingba G, Wachs J. Gestonurse: a robotic surgical nurse for handling surgical instruments in the operating room. J Robot Surg. 2012;6(1):53–63. PMID: 27637980

75. Pérez F, Sossa H, Martínez R, Lorias D. Video-based tracking of laparoscopic instruments using an orthogonal webcams system. Acad Sci Eng Technol Int J 2013;7(8):440-3. doi: 10.5281/zenodo.1086517

82. Grange S, Terrence W, Fong T, Baur C. M/ORIS: A medical/operating room interaction system. In: 6th International Conference on Multimodal Interfaces (ICMI) ACM. ACM; 2004. p. 159–66. doi:10.1145/1027933.1027962

100. Wachs J, Stern H, Edan Y, Gillam M, Feied C, Smith M, et al. A Real-Time Hand Gesture Interface for Medical Visualization Applications. In: Tiwari A, Roy R, Knowles J, Avineri E, Dahal K, editors. Applications of Soft Computing, volume 36 of Advances in Intelligent and Soft Computing. Springer Berlin Heidelberg.; 2006. p. 153–62. doi: https://doi.org/10.1007/978-3-540-36266-1_15
